# Transcranial Magneto-Acoustic Stimulation Attenuates Synaptic Plasticity Impairment through the Activation of Piezo1 in Alzheimer’s Disease Mouse Model

**DOI:** 10.34133/research.0130

**Published:** 2023-05-08

**Authors:** Fangxuan Chu, Ruxin Tan, Xin Wang, Xiaoqing Zhou, Ren Ma, Xiaoxu Ma, Ying Li, Ruixu Liu, Chunlan Zhang, Xu Liu, Tao Yin, Zhipeng Liu

**Affiliations:** ^1^ Institute of Biomedical Engineering, Chinese Academy of Medical Sciences and Peking Union Medical College, Tianjin, 300192, China.; ^2^ Tianjin Institutes of Health Science, Tianjin, 301600, China.; ^3^ Neuroscience Center, Chinese Academy of Medical Science & Peking Union Medical College, Beijing, 100730, China.

## Abstract

The neuropathological features of Alzheimer’s disease include amyloid plaques. Rapidly emerging evidence suggests that Piezo1, a mechanosensitive cation channel, plays a critical role in transforming ultrasound-related mechanical stimuli through its trimeric propeller-like structure, but the importance of Piezo1-mediated mechanotransduction in brain functions is less appreciated. However, apart from mechanical stimulation, Piezo1 channels are strongly modulated by voltage. We assume that Piezo1 may play a role in converting mechanical and electrical signals, which could induce the phagocytosis and degradation of Aβ, and the combined effect of mechanical and electrical stimulation is superior to single mechanical stimulation. Hence, we design a transcranial magneto-acoustic stimulation (TMAS) system, based on transcranial ultrasound stimulation (TUS) within a magnetic field that combines a magneto-acoustic coupling effect electric field and the mechanical force of ultrasound, and applied it to test the above hypothesis in 5xFAD mice. Behavioral tests, in vivo electrophysiological recordings, Golgi–Cox staining, enzyme-linked immunosorbent assay, immunofluorescence, immunohistochemistry, real-time quantitative PCR, Western blotting, RNA sequencing, and cerebral blood flow monitoring were used to assess whether TMAS can alleviate the symptoms of AD mouse model by activating Piezo1. TMAS treatment enhanced autophagy to promote the phagocytosis and degradation of β-amyloid through the activation of microglial Piezo1 and alleviated neuroinflammation, synaptic plasticity impairment, and neural oscillation abnormalities in 5xFAD mice, showing a stronger effect than ultrasound. However, inhibition of Piezo1 with an antagonist, GsMTx-4, prevented these beneficial effects of TMAS. This research indicates that Piezo1 can transform TMAS-related mechanical and electrical stimuli into biochemical signals and identifies that the favorable effects of TMAS on synaptic plasticity in 5xFAD mice are mediated by Piezo1.

## Introduction

Alzheimer’s disease (AD) is a complex brain disease characterized by the β-amyloid (Aβ) deposition, neurofibrillary tangle generation, neuroinflammation, and synaptic loss [1,2]. For the animal model of AD, 5xFAD mice show AD-like pathology in a certain sequence, Aβ deposition and microglial proliferation at 1.5 to 2 months of age, cognitive impairment occurs at 4 to 5 months of age, and neuron loss occurs by 9 months of age [3,4]. Microglia are highly mechanosensitive cells that monitor changes in the mechanical properties of the brain microenvironment in the context of neurodegenerative disorders [5]. Specifically, they mechanically and chemically interact with the brain microenvironment [6], which monitor neuronal activity, release pro- and/or anti-inflammatory mediators to regulate neuroinflammation, and clear abnormally aggregated proteins (e.g., Aβ) by phagocytic mechanisms to promote the repair of damage and the recovery of homeostasis in the brain [2,7]. However, in the course of AD, the balance is interrupted and dysfunctional microglia impair the clearance of Aβ as well as disrupt the homeostasis of the neuronal microenvironment, which further exacerbate the inflammatory response and AD progression [8]. Although the biophysical principles of microglia remain unclear, several studies have emphasized the increasing importance of modulating microglial mechanical sensitivity in the treatment of AD [5,8–10].

Piezo1, a mechanosensitive ion channel with a trimeric propeller-like structure, comprises 2,100 to 4,700 amino acids and 24 to 40 transmembrane domains [11]. This special structure allows Piezo1 to act as a mechanically gated cation channel that rapidly responds to various ways of mechanical stimuli and converts mechanical signals into biological signals to adjust different body functions [12,13]. Piezo1 might also perceive alterations in the mechanical stiffness of brain tissue; Velasco-Estevez et al. [14] found that the mechanosensitive ion channel Piezo1 is up-regulated in astrocytes surrounding inflexible Aβ fibrils. The latest research shows that the activation of Piezo1 with agonist Yoda1 can orchestrate Aβ clearance via enhancement of microglial phagocytosis and lysosomal activity and that astrocytic Piezo1 robustly regulates adult hippocampal neurogenesis, LTP (long-term potentiation), and cognitive function [8,10]. Rapidly emerging studies suggest that Piezo1 plays a dominant regulatory part in the mechanical conduction of ultrasound and can obviously mediate ultrasonic stimulation of neurons, tumor cells, and osteoblastic cells to trigger downstream cellular signal processes; for example, activation of Piezo1 by ultrasound can regulate neuronal function, inhibit tumor growth, and promote osteogenesis [15–17], highlighting the importance of Piezo1 in brain function and ultrasonic signal transduction. Microglia are important phagocytic and immune cells that regulate the pathological process of AD, but the importance of Piezo1-mediated mechanotransduction in microglia for brain functions is less appreciated.

Recently developed neuromodulatory techniques, such as electrical, magnetic, optical, and ultrasound stimulation, provide unprecedented opportunities to study brain function and treat brain diseases [18–21]. Transcranial magneto-acoustic stimulation (TMAS) is a type of new noninvasive electrical stimulation system established with transcranial ultrasound stimulation (TUS) and magnetic field, which could combine a magneto-acoustic electric field and the mechanical force of ultrasound. The conductive particles inside the brain tissue driven by ultrasound waves can stimulate the generation of electric field current in the magnetic field. The correlation between the electric field and the velocity of the particles enables manipulation of the effect of TMAS on the basis of Faraday’s law [22,23]. TMAS can achieve high spatial resolution at low millimeter level deep in the brain by using the high focusing property of ultrasound [22]. Compared with transcranial magnetic stimulation (TMS), TMAS can obtain better localization and target stimulation of functional brain areas while ensuring stimulation depth [23,24], which has important research value and application prospect especially in the field of functional brain partitioning and deep transcranial stimulation with higher requirements for stimulation targeting.

Interestingly, the Piezo1 ion channel is also strongly modulated through voltage [25,26]. Considering that Piezo1 is highly sensitive to mechanical and voltage stimulation, we assumed that Piezo1 expressing on the membrane of mechanosensitive microglia may be a crucial channel involving in mechanotransduction, play a role in converting TMAS-associated mechanical and electrical signals, and activate microglial autophagy to phagocytize and degrade Aβ through the activation of Piezo1.

## Results

### TMAS improved the cognitive function in 5xFAD mice through Piezo1

**Fig. 1.**
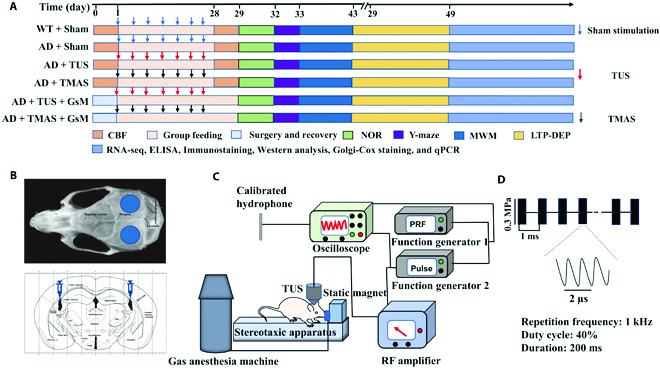
Description of the experimental processes and instruction of the treatment procedure. (A) Timeline of the experiment. (B) Schematic of the stimulation area and GsMTx-4 injection area [71]. (C) TMAS and TUS systems. (D) Schematic of the stimulation parameters.

**Fig. 2.**
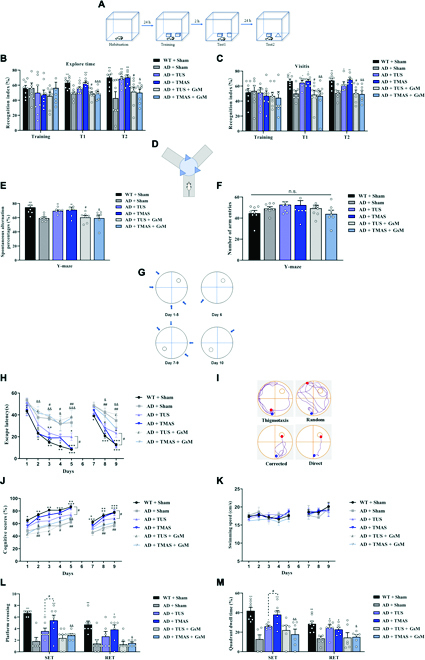
TMAS treatment alleviates cognitive disorder in 5xFAD mice. (A) Protocol of the NOR test. (B and C) The RI based on the exploration duration and visit frequency. (D) Protocol of the Y-maze test. (E) The spontaneous alternation percentages in the Y-maze test. (F) The number of arm entries in the Y-maze test. (G) Experimental protocol of the MWM test. (H) Escape latencies in the IT and RT phases. (I) Illustration of the search strategies. (J and K) Cognitive scores and swimming speed in the IT and RT phases. (L and M) The number of platform crossings and quadrant occupancy time on day 6. *n* = 8 per group. *P < 0.05, **P < 0.01, ***P < 0.001 vs. the AD + Sham group. #*P* < 0.05, ##*P* < 0.01 vs. the AD + TUS group. &*P* < 0.05, &&*P* < 0.01, &&&*P* < 0.001 vs. the AD + TMAS group. n.s., not significant; SET, spatial exploration test; RET, reversal exploration test.

After experimental design, TUS and TMAS treatments were performed (Fig. 1). The novel object recognition (NOR), Y-maze, and Morris water maze (MWM) tests were performed to test whether TMAS could improve learning and memory, and assess the role of Piezo1 in the effect of TMAS. Hippocampus-dependent short-term object recognition memory were evaluated by the NOR test (Fig. 2A). The statistical analysis of the recognition index (RI) according to time spent exploring each object was showed that the time spent exploring the novel object was obviously higher in the AD + TMAS group than in the AD + Sham group (test 1, *P* < 0.001; test 2, *P* < 0.01; Fig. 2B). Similar findings were obtained for the RI of the number of visits (Fig. 2C). However, after administration of GsMTx-4, the therapeutic effect of TMAS was significantly prevented in 5xFAD mice (test 1, *P* < 0.001; test 2, *P* < 0.05, Fig. 2B). Similar findings were observed in the AD + TUS and AD + TUS + GsM groups.

The Y-maze test can evaluate short-term spatial working memory (Fig. 2D). The spontaneous alternation percentage was noticeably improved in AD mice after TMAS, demonstrating that TMAS treatment was effective in alleviating spatial memory deficits (*P* < 0.01, Fig. 2E). However, this therapeutic effect of TMAS was blocked by GsMTx-4 (*P* < 0.05, Fig. 2E). Similar findings were found in the AD + TUS and AD + TUS + GsM groups. In addition, there was no difference in the total number of arm entries between the groups (*P* > 0.05, Fig. 2F).

The MWM test was performed to test spatial cognitive function [27]. Two-way analysis of variance showed visible differences in escape latency between and within groups for each day (*P* < 0.001). Post hoc analysis indicated that escape latency was longer in the AD + Sham group compared to the WT (wild-type) + Sham group (*P* < 0.001, Fig. 2H), and escape latency was shorter in AD + TMAS group than in the AD + Sham group (*P* < 0.001, Fig. 2H). However, this effect of TMAS was prevented by GsMTx-4 administration. Furthermore, we analyzed the cognitive scores of the mice based on their swimming pathway (Fig. 2I); similar findings were observed in cognitive scores (Fig. 2J). Interestingly, there were visible differences in cognitive score and escape latency on the last days of the initial training (IT) and reversal training (RT) phases between the AD + TMAS and AD + TUS groups (*P* < 0.05, Fig. 2H and J). Learning flexibility was evaluated in the RT stage, and similar findings were observed in RT phase (Fig. 2H and I). However, TUS and TMAS did not influence the swimming speed of MWM (Fig. 2K). In the spatial exploration test phase, the number of platform crossings and dwell time in the target quadrant were higher in the AD + TMAS group compared to the AD + Sham group (*P* < 0.001; Fig. 2I and M) and even higher than the AD + TUS group (*P* < 0.05, Fig. 2I and M). However, the therapeutic effects of TMAS were suppressed by GsMTx-4 administration. Similar findings were observed in reversal exploration test phase (Fig. 2I and M). Collectively, these behavioral findings indicate that TMAS can ameliorate cognitive impairment in 5xFAD mice to a greater extent than TUS and that Piezo1 plays a key role in TMAS-induced learning and memory formation.

### TMAS alleviated hippocampal synaptic plasticity impairment through Piezo1 in AD mouse model

**Fig. 3.**
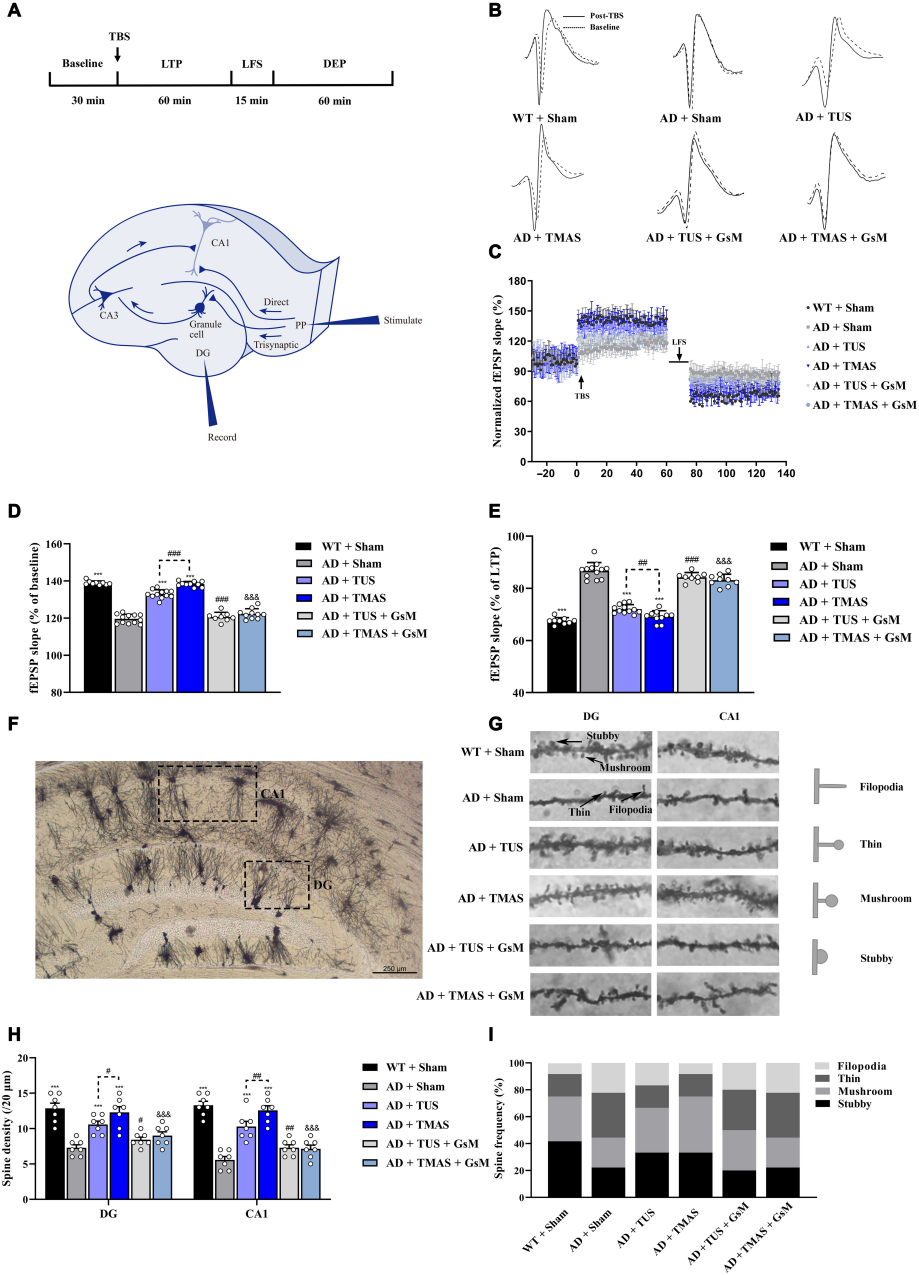
TMAS treatment ameliorates synaptic plasticity damage in 5xFAD mice. (A) Experimental protocol of in vivo electrophysiological recordings and positioning of stimulating and recording electrodes in hippocampal perforant pathway (PP)-DG circuit. (B) The fEPSP curve before and after LTP induction. (C) Changes in fEPSP slope from the PP to the DG. (D and E) Average fEPSP slope between the last 15 min during LTP and DEP. (F) Micrographs of Golgi–Cox-stained dendritic spines in the hippocampal area. Scale bar, 250 μm. (G) Representative picture of dendritic spines in the DG and CA1 region. The different types of dendritic spines are indicated by the black arrows. (H) The density of dendritic spines. (I) The proportions of different spine types. *n* = 8 per group. ***P < 0.001 vs. the AD + Sham group. #*P* < 0.05, ##*P* < 0.01, ###*P* < 0.001 vs. the AD + TUS group. &&&*P* < 0.001 vs. the AD + TMAS group. TBS, theta burst stimulation.

Synaptic plasticity, including LTP and depotentiation (DEP), is the biological mechanism underlying cognition [1]. In vivo electrophysiological recordings results found that the field excitatory postsynaptic potential (fEPSP) slope was decreased in the AD + Sham group compared with the WT + Sham group after theta burst stimulation (*P* < 0.001, Fig. 3B to D), illustrating that synaptic plasticity was damaged in 5xFAD mice. After TMAS treatment, 5xFAD mice showed a significant increase in LTP and a significant decrease in DEP (LTP and DEP: *P* < 0.001, Fig. 3C to E). TMAS was more effective than TUS in improving LTP and DEP in 5xFAD mice (*P* < 0.001, *P* < 0.01, Fig. 3D and E). In the AD + TUS + GsM and AD + TMAS + GsM groups, the effects of TUS and TMAS in ameliorating synaptic plasticity impairment were inhibited by GsMTx-4 administration (*P* < 0.001, Fig. 3D and E).

The dendritic spines of neurons are closely associated with synaptic plasticity [28]. Results showed that the dendritic spine density of hippocampal neurons in the dentate gyrus (DG) and corno ammonis 1 (CA1) regions in the 5xFAD mice was significantly increased after TMAS treatment (DG and CA1: *P* < 0.001, Fig. 3G and H). Interestingly, the density of dendritic spines in 5xFAD mice was higher after TMAS treatment than after TUS treatment (DG: *P* < 0.05; CA1: *P* < 0.01, Fig. 3H). Nevertheless, GsMTx-4 administration markedly suppressed the increase in dendritic spines density induced by TUS and TMAS treatment (DG: *P* < 0.05, *P* < 0.001; CA1: *P* < 0.01, *P* < 0.001, Fig. 3H). We also compared the proportions of the 4 spine types (filopodia, thin, mushroom, and stubby) in all groups (Fig. 3G and I). The proportion of stubby (mature) spines was obviously increased in the AD + TUS (33.3%, *P* < 0.001) and AD + TMAS groups (33.3%, *P* < 0.001) respectively. After TUS and TMAS treatment, the levels of synaptic plasticity-associated proteins (presynaptic vesicle membrane protein [SYP], postsynaptic scaffold protein [PSD-95], NR2B, NR2A, and dendritic spine development-regulating protein [DBN]) in the hippocampi and cortices of 5xFAD mice were enhanced (Fig. S1). TMAS treatment was better than TUS in improving the levels of synaptic related proteins, whereas GsMTx-4 administration suppressed the increase in synaptic-related proteins induced by TUS and TMAS treatment (Fig. S1). Together, these results demonstrate that TMAS ameliorates hippocampal synaptic plasticity impairment in 5xFAD mice and has a stronger effect than TUS and that the Piezo1 plays a critical role in regulating TMAS-induced neuronal plasticity.

### TMAS activated Piezo1 on microglia in the brain of 5xFAD mice

**Fig. 4.**
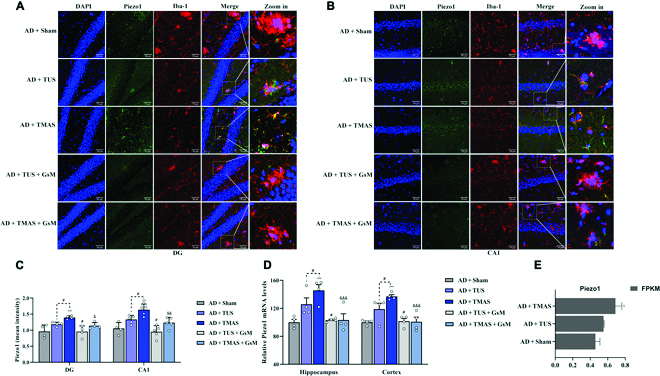
TMAS treatment activates Piezo1 in brain microglia. (A and B) Immunofluorescence images of Piezo1 and Iba-1 in the DG and CA1 regions. Scale bar, 50 μm. (C) The average fluorescence intensity of Piezo1. (D) The mRNA levels of piezo1 in hippocampal and cortical tissues. (E) RNA-seq analysis of Piezo1 in the hippocampus. *n* = 4 per group. *P < 0.05, ***P < 0.001 vs. the AD + Sham group. #*P* < 0.05 vs. the AD + TUS group. &*P* < 0.05, &&*P* < 0.01, &&&*P* < 0.001 vs. the AD + TMAS group.

We have assumed that Piezo1 could play a role in converting mechanical and electrical signals of TMAS; then, we next focused on whether TMAS could activate Piezo1 in the brain of 5xFAD mice. Immunostaining, real-time quantitative polymerase chain reaction (RT-qPCR), and RNA sequencing (RNA-seq) revealed that Piezo1 was expressed in the hippocampus and in microglia in 5xFAD mice (Fig. 4). We next tested whether Piezo1 channels in the brains of 5xFAD mice respond to 2 types of stimulation: TUS and TMAS. We performed costaining for Iba-1 and Piezo1, and we found that Piezo1 was expressed in microglia in the hippocampi of 5xFAD mice (Fig. 4A and B). Surprisingly, TMAS up-regulated the expression of Piezo1 in microglia in the hippocampus (DG and CA1: *P* < 0.001, Fig. 4C), but this up-regulatory effect of TMAS was prevented by GsMTx-4, an antagonist of Piezo1 (DG: *P* < 0.05; CA1: *P* < 0.01, Fig. 4C), and TMAS activated Piezo1 more significantly in the hippocampus of AD mice compared with in AD + TUS group (DG and CA1: *P* < 0.05, Fig. 4C). The similar results were obtained in RT-qPCR and RNA-seq (Fig. 4D and E). In addition, we also detected that the expression of Piezo1 in hippocampal neurons and TMAS also up-regulated its level (Fig. S2).

### TMAS promoted microglial migration and phagocytosis in vitro through the activation of Piezo1

**Fig. 5.**
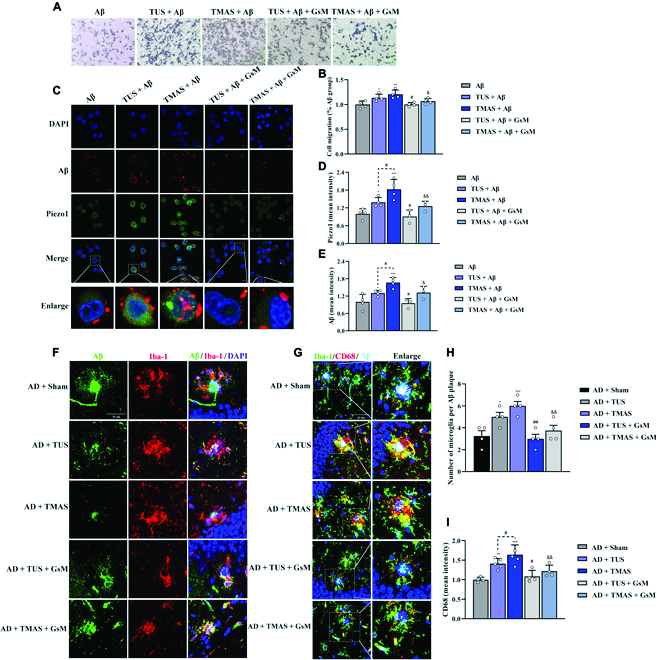
TMAS treatment promotes microglial migration and phagocytosis of Aβ through the activation of Piezo1. (A) Microglial migration was evaluated by the Transwell assays. Scale bar, 100 μm. (B) Graph showing microglial migration. (C) Representative immunofluorescence image of Aβ (red) and Piezo1 (green) in BV2 cells. Scale bar, 20 μm. (D and E) The average fluorescence intensity of Piezo1 and Aβ in BV2 cells. (F) Immunofluorescence images of Aβ and Iba1 in the brains of mice. (G) Immunofluorescence images of Iba1, CD68, and Aβ in the brains of mice. (H) Number of microglia in the vicinity of Aβ plaques. (I) The mean fluorescence intensity of CD68. *n* = 4 per group. *P < 0.05, **P < 0.01, ***P < 0.001 vs. the Aβ group and AD + Sham group. #*P* < 0.05, ##*P* < 0.01 vs. the TUS + Aβ group and AD + TUS group. &*P* < 0.05, &&*P* < 0.01 vs. the TMAS + Aβ group and AD + TMAS group.

With AD progression, microglia may exhibit decreased migration and phagocytosis and the inability to regulate damage and repair [29]. Mechanoreceptors are widely distributed in the brain, especially in microglia [5,30]. Due to their unique characteristics and the fact that they express the mechanosensor Piezo1, they may be a suitable mechano-monitoring system for accurately monitoring dynamic changes in the mechanical performance of the brain [5]. To investigate whether TMAS can activate Piezo1 to alter microglial functions that are vital for Aβ clearance. The Transwell assay revealed that the number of migrating BV2 cells (mouse microglia cells) was higher in the TMAS + Aβ group compared to Aβ group (*P* < 0.01, Fig. 5A and B). Similar results were found in the TUS + Aβ group. However, the effects of TMAS and TUS were dramatically diminished by GsMTx-4 (*P* < 0.05, Fig. 5A and B). After migrating to the pathological site, microglia maintain brain homeostasis by eliminating pathological particles (such as Aβ) by phagocytosis. Immunofluorescence with an anti-Piezo1 antibody indicated that Piezo1 was contained in BV2 cells and found in the plasma membrane and nucleus (Fig. 5C). After TMAS, the level of Piezo1, especially in the plasma membrane, was increased, and Aβ was gradually phagocytosed by BV2 cells (*P* < 0.001, Fig. 5C to E). Compared with TUS, TMAS was more effective in activating Piezo1 and inducing the phagocytosis of Aβ (*P* < 0.05, Fig. 5C to E), and Aβ was also present into the nucleus. However, this ability of TMAS and TUS to induce phagocytosis was blocked by GsMTx-4. Aβ plaques resemble hard foreign objects (Aβ plaques: 3 × 10^9^ Pa; normal brain tissue: 200 to 500 Pa) that represent a change in the extrinsic mechanical environment in vivo [8], and Piezo1 channels in microglia can potentially regulate mechanotransduction in response to Aβ and attempt to phagocytose them [14,31]. Therefore, it is not amazing that microglia surrounding Aβ plaques display a marked enhance in Piezo1 expression (Fig. 5C and F), and Piezo1 could act as an important mechanical and electrical stimulus-sensing molecule in the process during TMAS by promoting the migration and phagocytic activity of BV2 cells.

### TMAS mediated microglial phagocytosis of Aβ in vivo through Piezo1

Since TMAS induced activation of Piezo1 promotes microglial phagocytosis in vitro, we next studied the effect of TMAS in 5xFAD mice. First, we demonstrated that Piezo1 channels were expressed in the brains of 5xFAD mice, albeit at low levels, and that TMAS significantly up-regulated the expression of Piezo1 (Fig. 4). Confocal microscopy displayed that microglia were attracted to the vicinity of Aβ plaques in 5xFAD mice (Fig. 5F and H). TMAS treatment obviously recruited more microglia to do more phagocytosis of Aβ and decreased Aβ deposition in the brains of 5xFAD mice (*P* < 0.05, *P* < 0.001, Fig. 5F and H). In addition, we evaluated the expression levels of CD68 (Fig. 5G), a lysosomal marker that indicates the presence of microglial phagosomes, and the occurrence of Aβ internalization and degradation. The fluorescence intensity of CD68 was enhanced in the AD + TMAS group compared with the AD + Sham group (*P* < 0.001, Fig. 5I), and the effect of TMAS was stronger than that of TUS (*P* < 0.05, Fig. 5I). However, fewer microglia were recruited to Aβ plaques, and CD68 was expressed at lower levels in the Aβ + TUS + GsM and Aβ + TMAS + GsM groups, revealing that the effects of TUS and TMAS in promoting phagocytosis were inhibited by an antagonist of piezo1 (GsMTx-4) (microglial number: *P* < 0. 01, *P* < 0.001; CD68 intensity: *P* < 0.05, *P* < 0.01 Fig. 5H and I). Immunostaining and enzyme-linked immunosorbent assay indicated that the levels of Aβ in the hippocampus and cortex were decreased in 5xFAD after TMAS and TUS treatment (Fig. S3) and that TMAS treatment had a stronger effect than TUS. However, the effects of TMAS and TUS in decreasing Aβ levels in the brains of 5xFAD mice were inhibited by GsMTx-4. These results suggest that TMAS treatment can activate Piezo1, which may promote more microglial recruitment to the vicinity of Aβ plaques to do more phagocytosis, thus facilitating Aβ clearance and maintaining brain homeostasis in 5xFAD mice.

### TMAS activated autophagy in the brains of 5xFAD mice through Piezo1

**Fig. 6.**
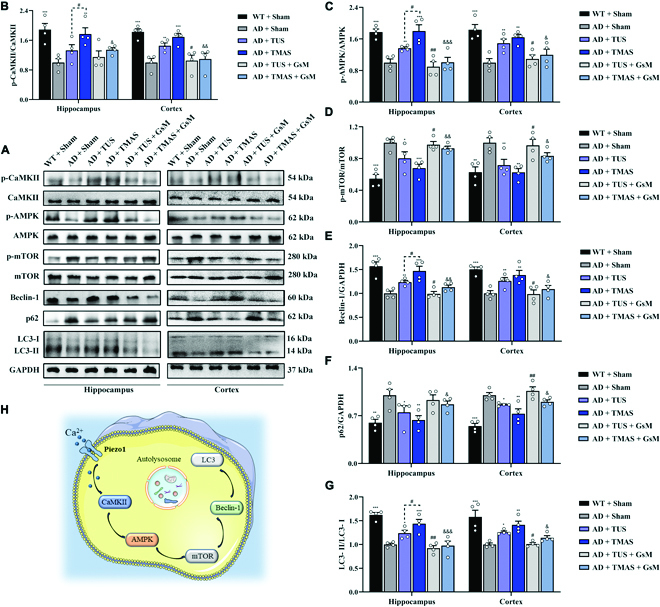
TMAS treatment activates autophagy via the CaMKII/AMPK/mTOR signaling pathway. (A) Immunoblotting of p-CaMKII, CaMKII, p-AMPK, AMPK, p-mTOR, mTOR, Beclin-1, p62, and LC3 in the hippocampus and cortex. (B to G) Quantitative analysis of p-CaMKII/CaMKII (B), p-AMPK/AMPK (C), p-mTOR/mTOR (D), Beclin-1/glyceraldehyde-3-phosphate dehydrogenase (GAPDH) (E), p62/GAPDH (F), and LC3-II/LC3-I (G) levels in the hippocampus and cortex. (H) Schematic diagram of CaMKII/AMPK/mTOR signaling pathway-mediated activation of Piezo1 channels induced by TMAS. *n* = 4 per group. *P < 0.05, **P < 0.01, ***P < 0.001 vs. the AD + Sham group. #*P* < 0.05, ##*P* < 0.01 vs. the AD + TUS group. &*P* < 0.05, &&*P* < 0.01, &&&*P* < 0.001 vs. the AD + TMAS group.

In AD brains, the deposition of Aβ is phagocytosed by microglia and then degraded by autodegradation systems such as autophagy [32]. With these facts, we next assessed the modulatory of TMAS on autophagy in the brains of AD mice. Piezo1 can regulate the brain vasculature, immune responses, neural stem cell differentiation, synaptic transmission, and macrophage polarization by calmodulin-dependent protein kinase II (CaMKII) [33–36]. Activation of CaMKII can induce autophagy via phosphorylation of adenosine monophosphate-activated protein kinase (AMPK) and further suppression of mammalian target of rapamycin (mTOR), resulting in the generation of autophagosomes [37,38]. Microtubule-associated light chain 3 (LC3) is an autophagy-associated protein that increases in the LC3-II to LC3-I ratio indicates the formation of autophagosomes. Autophagosomes and lysosomes converge to form autolysosomes, in which autophagy adaptor p62 (p62) is degraded (Fig. 6H) [38,39]. The levels of p-CaMKII, p-AMPK, and Beclin-1 and the LC3-II/LC3-I ratio were elevated, and the levels of p-mTOR and p62 were reduced in the hippocampi of AD mice after TMAS treatment (p-CaMKII/CaMKII, *P* < 0.01; p-AMPK/AMPK, *P* < 0.001; p-mTOR/mTOR, *P* < 0.001; Beclin-1, *P* < 0.001; p62, *P* < 0.01; LC3-II/LC3-I, *P* < 0.001, Fig. 6A to G). Similar findings were observed in the cortices of AD mice after TMAS treatment. Furthermore, the regulation effect of TMAS on the level of autophagy associated proteins was stronger than that of TUS (p- CaMKII/CaMKII, *P* < 0.05; p-AMPK/AMPK, *P* < 0.05, Beclin-1, *P* < 0.05, Fig. 6B to E). However, the Piezo1 antagonist GsMTx-4 blocked the increased level of autophagy induced by TMAS and TUS (Fig. 6A to G). Microglial and neuronal autophagy levels were evaluated by immunofluorescence. The expression of LC3 in microglia in the DG and CA1 regions of the hippocampus was increased in the AD + TMAS group (Fig. S4). These results suggest that activation of Piezo1 channels is enough to trigger autophagy in 5xFAD mice through the CaMKII/AMPK/mTOR signaling pathway and that TMAS can increase CaMKII phosphorylation through Piezo1, in turn increasing autophagy levels in AD mice.

### TMAS treatment alleviated neuroinflammation in 5xFAD mice through Piezo1

**Fig. 7.**
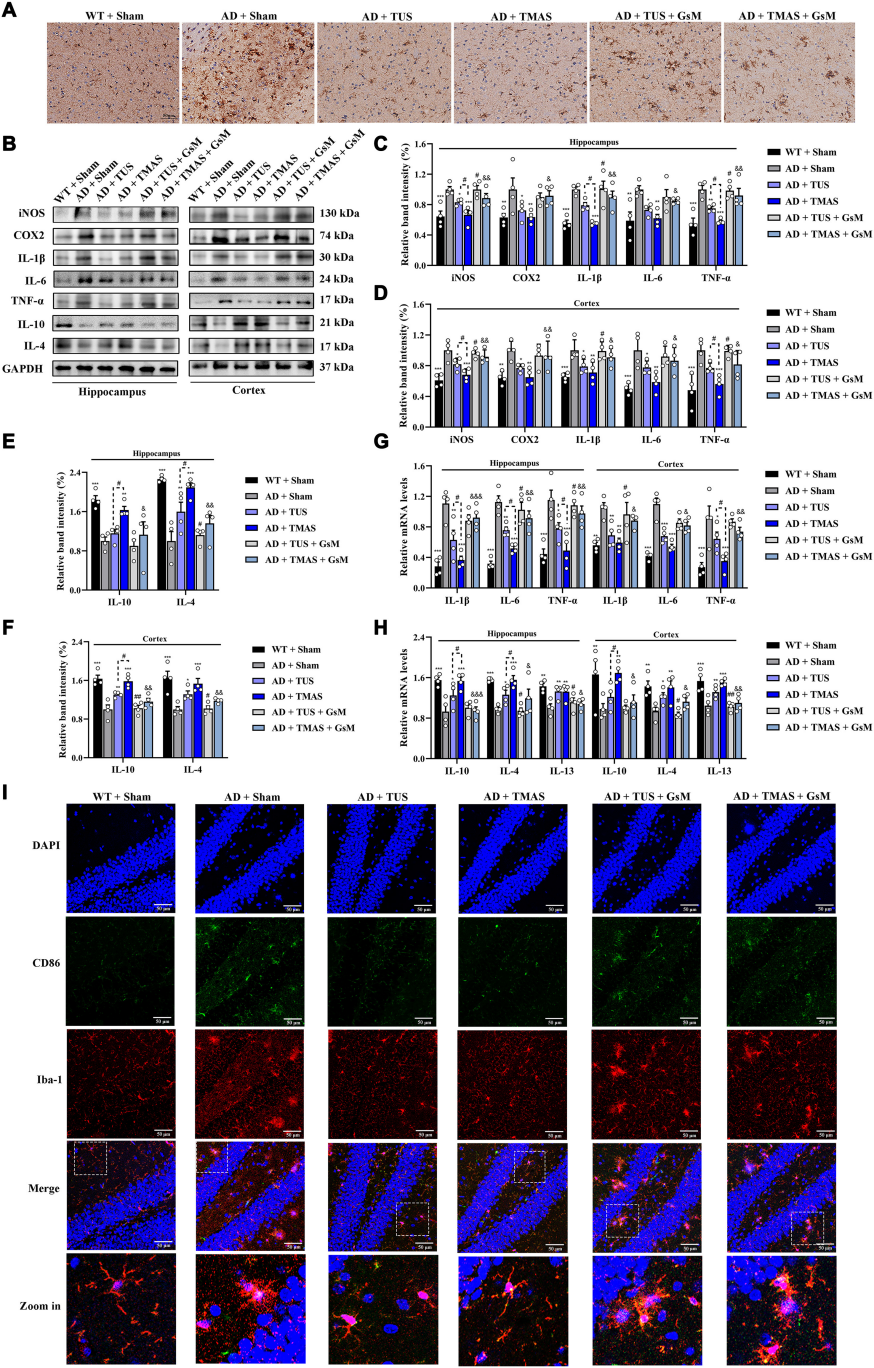
TMAS treatment alleviates the neuroinflammation in 5xFAD mice. (A) Immunohistochemical of Iba1 in the brains of mice. Scale bar, 50 μm. (B) Immunoblotting of iNOS, COX-2, IL-1β, IL-6, TNF-α, IL-10, and IL-4 in the hippocampus and cortex. (C and D) TMAS inhibited the production and release of proinflammatory factors in the hippocampi and cortices of 5xFAD mice. (E and F) TMAS increased the protein levels of IL-4 and IL-10 in the brain of 5xFAD mice. (G and H) TMAS inhibited the mRNA expression of proinflammatory factors and increased the mRNA expression of anti-inflammatory factors in the hippocampi and cortices of 5xFAD mice. (I) Fluorescence images of CD86 and Iba1 in the DG area. Scale bar, 50 μm. *n* = 4 per group. *P < 0.05, **P < 0.01, ***P < 0.001 vs. the AD + Sham group. #*P* < 0.05 vs. the AD + TUS group. &*P* < 0.05, &&*P* < 0.01, &&&*P* < 0.001 vs. the AD + TMAS group.

Microglial autophagy-related phagocytosis is crucial for the alleviation of neuroinflammation [40]. We next investigated whether TMAS treatment can reduce neuroinflammation through Piezo1 in 5xFAD mice. Immunohistochemistry with an antibody against the microglia-specific marker Iba1 indicated that overactivated microglia were present in the brains of 5xFAD mice and that the number of overactivated microglia was decreased through TUS and TMAS treatment (Fig. 7A). However, the effects of TUS and TMAS were inhibited by GsMTx-4. Furthermore, the levels of proinflammatory cytokines, including inducible nitric oxide synthase (iNOS), cyclooxygenase-2 (COX-2), interleukin-1β (IL-1β), interleukin-6 (IL-6), and tumor necrosis factor-α (TNF-α) were obviously decreased (Fig. 7B to H), while the levels of anti-inflammatory cytokines, containing interleukin-4 (IL-4) and interleukin-10 (IL-10), in the hippocampi and cortices of 5xFAD mice were increased after TMAS treatment (Fig. 7B to H) but suppressed in the AD + TMAS + GsM group. TMAS had a stronger effective than TUS in reducing the number of microglia and modulating the levels of pro- or anti-inflammatory cytokines. Microglia display M1 and M2 phenotypic characteristics according to whether they express pro- or anti-inflammatory factors [41], and overactivation of M1 microglia accelerates to the spread of Aβ, which in turn promotes the differentiation of microglia to the M1 phenotype, leading to an imbalance in microglia polarization and the development of AD [42,43]. To analyze the effects of TMAS on microglial polarization, we conducted double labeling for Iba-1 and CD86 or CD206 (an M1 microglia marker or an M2 microglia marker) (Fig. 7I and Fig. S5). The results found that the level of CD86 in microglia was enhanced in the DG and CA1 regions of the hippocampus in AD mice and that microglia were hyperactivated, as they exhibited a hypertrophic amoeboid shapes with shortened and thickened processes. TMAS decreased the level of CD86, increased the level of CD206, and normalized microglial morphology in the hippocampal DG and CA1 regions in 5xFAD mice (Fig. 7I and Fig. S5). However, the effect of TMAS on microglia was blocked by GsMTx-4. These results show that TMAS treatment effectively alleviates microglia-related chronic neuroinflammation through Piezo1 and that the effect of TMAS was stronger than that of TUS.

### Hippocampal transcriptome analysis revealed the beneficial effect of TMAS in 5xFAD mice involved Piezo1 activation

**Fig. 8.**
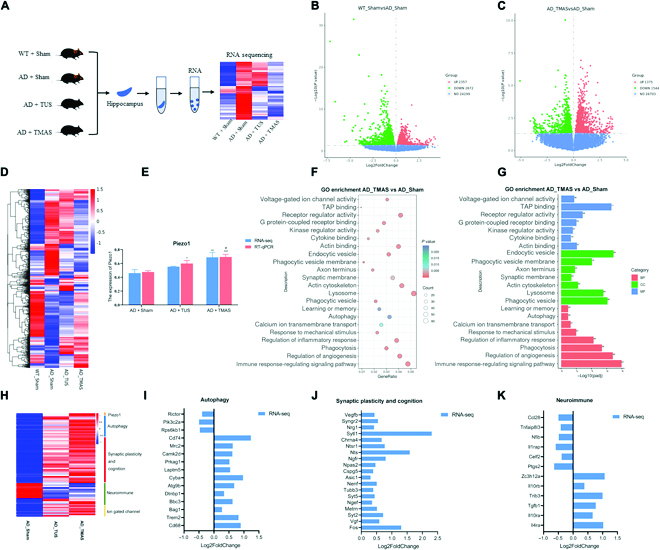
Transcriptome analysis of hippocampal tissue reveals that TMAS exerts its beneficial effect by activating Piezo1 in 5xFAD mice. (A) Schematic of RNA-seq analysis of hippocampal tissue from the 4 groups. (B and C) Differentially expressed genes compared to 5xFAD mice (log2 fold change > 0, *P* < 0.05). (D) Heatmap shows differentially expressed genes of 4 groups (log2 fold change > 0, *P* < 0.05). (E) The bar graph shows the expression of Piezo1, as determined by RT-qPCR and RNA-seq (*P* < 0.05). (F and G) GO enrichment analysis of differentially expressed genes in the brains of AD mice after TMAS treatment (log2 fold change > 0, *P* < 0.05). (H) Heatmap showing the relative gene expression of Piezo1- and autophagy-, synaptic plasticity-, and neuroimmunity-related genes in the AD, TUS, and TMAS-treated groups (log2 fold change > 0, *P* < 0.05). (I to K) The fold change in the expression of genes related to autophagy, synaptic plasticity, and neuroimmunity after TMAS treatment, as determined by RNA-seq (log2 fold change > 0, *P* < 0.05). *n* = 5 per group. BP, biological process; CC, cellular component; MF, molecular function.

To further investigate the mechanism underlying the effect of TMAS on cognitive function and synaptic plasticity in AD mice, we isolated hippocampal tissue from mice in the 4 groups and performed RNA-seq analysis (Fig. 8A). We identified 2,357 up-regulated genes and 2,672 down-regulated genes in the WT + Sham group compared with the AD + sham group and 1,375 up-regulated genes and 1,544 down-regulated genes in the AD + TMAS group compared to the AD + Sham group (Fig. 8B and C). Hierarchical clustering analysis of hippocampal tissue from TMAS- and TUS-treated 5xFAD mice also revealed differences in the brain transcriptome (Fig. 8D). Notably, TMAS significantly increased Piezo1 expression in the hippocampi of 5xFAD mice (RNA-seq: *P* < 0.01; RT-qPCR: *P* < 0.001, Fig. 8E) and had a stronger effect in activating piezo1 than TUS (RNA-seq: *P* = 0.083 > 0.05; RT-qPCR: *P* = 0.045 < 0.05, Fig. 8E). Then, we conducted gene ontology (GO) analysis of the differentially expressed genes in the AD + TMAS group (Fig. 8F). GO analysis showed that the differentially expressed genes were significantly enriched in lysosome, phagocytosis and autophagy, calcium ion transport, angiogenesis, synapses, learning and memory, as well as immune and inflammatory responses; genes associated with mechanical stimulation and voltage-sensitive ion channels were also enriched (Fig. 8F). The differentially expressed genes were also associated enriched with in the biological process, cellular component, and molecular function (Fig. 8G), suggesting that TMAS can improve cognition through autophagy and immunoregulation. The heatmap of gene expression also demonstrated that TMAS and TUS treatment significantly up-regulated and/or down-regulated genes associated with autophagy, synaptic plasticity, and neuroimmunity and some ion gated channels, such as Piezo1 (Fig. 8H). TMAS appeared to have a stronger effect in regulating the expression of these genes than TUS (Fig. 8H). The bar graph in Fig. 8I to K displays the fold change in the level of genes shown to be included in autophagy, synaptic plasticity, and neuroimmunity in Fig. 8H in the AD + TMAS group. RNA-seq analysis of hippocampal tissue revealed that TMAS not only up-regulated the expression of Piezo1 in the hippocampi of 5xFAD mice but also altered the level of autophagy-, immune response-, and synaptic plasticity-related genes in 5xFAD mice.

## Discussion

Here, we designed a TMAS system that combines a magneto-acoustic coupling effect electric field and the mechanical force of ultrasound. We found that Piezo1 acts as an important transducer of TMAS-related mechanical and electrical stimuli to alleviate AD. Up-regulation of Piezo1 expression by TMAS resulted in the activation of microglial autophagy, leading to the phagocytosis and degradation of Aβ (Graphical abstract). Furthermore, TMAS regulated LTP, synaptic plasticity, hippocampus-dependent cognitive function, microglial M1/M2 polarization, inflammatory cytokine levels, and brain rhythms in 5xFAD mice through Piezo1 and had a stronger effect than TUS. Moreover, inhibition of Piezo1 by GsMTx-4 administration obviously suppressed the beneficial effects of TMAS. Our work not only shows that TMAS is advantageous for the treatment of brain aging and neurodegenerative diseases but also provides new insight into the mechanism by which Piezo1 transforms TMAS-related mechanical and electrical stimuli into biochemical signals and identifies Piezo1 as a novel therapeutic target for AD.

TMAS is a noninvasive focused electrical nerve stimulation technique with a good stimulation focus and stimulation depth. The stimulation effect could be controlled by adjusting the ultrasound intensity owing to the mechanism by which the electric field is generated through ultrasound within the magnetic field [44]. We have demonstrated that TMAS can be used to generate an electric field in a conductive medium upright to the orientation of the magnetic and sound fields and that the electric field coverage is extremely consistent with that of the acoustic field in our previous work [23]. TMAS integrates a magneto-acoustic electric field and the mechanical force of TUS [22]. The mechanical force of TUS may alter ion channels so that electric field stimulation has a much stronger effect. On the other hand, the electrical field affects the local potential or the ion channel threshold, and ultrasound can also exert a stronger effect in this way. TMAS combines a magneto-acoustic coupling effect electric field and the mechanical force of ultrasound, and whether it can further activate microglial Piezo1 and regulate the function of microglia surrounding Aβ, particularly their ability to clear Aβ by phagocytosis, may be vital for whether it can control the pathological processes of AD. To determine the role of TUS in the TMAS effect, mice were treated with TUS. The mRNA level of Piezo1 in the hippocampi of 5xFAD mice was evaluated by RNA-seq and RT-qPCR (Fig. 8E), and it was found that Piezo1 was expressed and localized on microglial plasma membrane and nucleus (Fig. 5) and that TMAS can further up-regulate the expression of Piezo1.

We discovered that Piezo1 plays a vital role in the TMAS-mediated increase in microglial migration and phagocytosis by acting as a sensor of mechanical and electrical stimuli (Fig. 5). TMAS further up-regulated the level of Piezo1 in microglia and promoted microglial phagocytosis of Aβ in vitro and in vivo through Piezo1 (Fig. 5C and F), showing a stronger effect than TUS. Along with Piezo1 expression, the expression of autophagy-, lysosome-, and phagocytosis-related genes was up-regulated in the hippocampus of the AD + TMAS group (Figs. 5, 6, and 8). However, these effects of TMAS were blocked after GsMTx-4 administration. These findings illustrate that TMAS promotes the phagocytosis of Aβ via the autophagy-lysosomal system by activating Piezo1 in 5xFAD mice. Piezo1-induced activation of CaMKII not only enhances the phagocytic activity of macrophages but also increases the expression of c-Fos and p-CREB in neurons [16,45]. Activation of CaMKII can also induce autophagy via p-AMPK and suppression of mTOR, leading to the formation of autophagosomes [38,46]. We found that Ca^2+^ transmembrane transport and CaMKII were up-regulated and enriched in the hippocampus of TMAS-treated AD mice (Figs. 6B and 8F to I). AMPK and mTOR were also participated in this process (Fig. 6A to D), and these effects of TMAS were inhibited after GsMTx-4 administration. These data showed that TMAS can regulate the CaMKII/AMPK/mTOR pathway by activating Piezo1 and then promote the phagocytosis and degradation of Aβ by autophagy in 5xFAD mice.

The activation of Piezo1 provides a driving force for calcium-dependent phagocytosis, which is crucial for activating crucial for activating the normal protective functions of microglia, including immune surveillance of the brain and regulation of neuroinflammation [8,47]. We also observed that TMAS treatment obviously inhibited overactivation of microglia, regulated the proinflammatory M1 and anti-inflammatory M2 polarization of microglia (Fig. 7I and Fig. S5), significantly reduced the levels of inflammatory cytokines, and increased the levels of anti-inflammatory factors in the hippocampi and cortices of 5xFAD mice (Fig. 7B to H). However, the anti-inflammatory effect of TMAS was inhibited by GsMTx-4, suggesting that TMAS exerted anti-inflammatory effects by activating Piezo1. Excessive accumulation of Aβ results in excessive microglial activation and neuroinflammation, whereas activation of autophagy usually alleviates of neuroinflammation by inducing the degradation of Aβ [48,49]. It makes sense that TMAS therapy alleviates aging-related inflammation by enhancing autophagy and normalizing microglial function.

Piezo1-mediated mechanotransduction evidently increases hippocampal neurogenesis, axonal growth, and LTP in the brain and cognitive functions [10,50]. Our data showed that TMAS treatment significantly improved bidirectional synaptic plasticity (LTP and DEP), increased dendritic richness and spine density (Fig. 3), and elevated the levels of the synapse-associated proteins in 5xFAD mice (Fig. S1); however, these changes did not occur after pretreatment with the Piezo1 inhibitor GsMTx-4. RNA-seq analysis also showed that the genes that were up-regulated in the hippocampus in the AD+TMAS group were enriched in learning and memory, the synaptic membrane, and the axon terminus (Fig. 8F, G, and J). In this study, we conducted several behavioral tests (the NOR, Y-maze, and MWM test) to test learning and memory of mice. The data showed that TMAS treatment increased the RI, spontaneous alternation percentage, number of platform crossings, and dwell time in the target region as well as reduced the escape latency of 5xFAD mice (Fig. 2), that TMAS had a stronger effect than TUS in improving cognitive function and alleviating synaptic plasticity impairment, and that the effect of TMAS was blocked by GsMTx-4. These results suggest that TMAS can ameliorate AD-related synaptic plasticity impairment and cognitive deficits through activation of Piezo1.

Apart from improvements in molecular and pathological changes, we discovered electrophysiological results of cognitive improvement, including enhanced hippocampal gamma band power, phase locking value, and phase-amplitude coupling following TMAS treatment (Fig. S6). Theta and gamma oscillations in neural networks play a crucial part in learning and memory, such as the distribution of attention and the storage of information [4,51,52]. Alterations in gamma have also been found in various brain regions in several neurological and brain aging diseases, including decreased spontaneous gamma synchronization in AD patients and decreased gamma power in several AD mouse models [53,54]. Various noninvasive brain stimulation techniques can modulate neural responses and cognitive flexibility by entraining gamma. The ultrasound-based gamma-band entrainment technique and transcranial alternating current stimulation at gamma frequency reduced the Aβ load and modulated brain rhythm in AD mice [52,54]. Some neuromodulation techniques (ultrasound, 1 kHz; rTMS, 20 Hz; etc.) can also modulate theta and gamma rhythms in the mouse hippocampus without gamma entrainment [4,55]. We also found that TMAS increased the gamma power in our study (Fig. S6B and C). TMAS is a compound electrical stimulation with superimposed magnetic and acoustic fields. It is worth exploring whether TMAS entrainment of gamma is a treatment modality for AD in the future. Furthermore, increasing evidence indicates that phase coupling promotes information transmission in neural networks and may connect functionally associated areas of the brain [56]. We discovered that the coupling of theta and gamma oscillations in the DG region was enhanced in 5xFAD mice after TMAS treatment (Fig. S6D to G). The regulation of cerebral blood flow (CBF) is crucial for maintaining normal brain function. In neurodegenerative disorders such as AD, CBF dysregulation and neurovascular malfunction occur prior to cognitive decline and hippocampal atrophy [57]. Results revealed that TMAS targeting the hippocampus could enhance the CBF in AD mice (Fig. S6H and I).

Most neuromodulation studies use mechanical pressure below 0.6 MPa when studying the brain [58,59]. Noninvasive ultrasound can activate the mechanosensitive ion channels (MscL-G22S) and trigger neural activation in mice at 0.3-MPa sound pressure [60]. Noninvasive ultrasonic neuromodulation of neuronal excitability for treatment of epilepsy in epileptic monkey models at 0.35-MPa sound pressure [61]. In addition, according to the stimulus pulse train, acoustic pressure (0.3 MPa), and duty cycle (Fig. 1D), we also calculated that *I_sppa_* and *I_spta_* were 3 and 1.2 W/cm^2^, respectively. The values of *I_sppa_* and *I_spta_* were within the stimulation range that had an excitatory effect on the neurons of mice [62]. The acoustic pressure of 0.3 MPa can also activate neurons in vitro by opening the Piezo1 channel and increasing the levels of p-CaMKII, p-CREB, and c-Fos [16]. According to theoretical calculations $\left(E=\frac{1}{\mathrm{\rho Cs}}PB\right)$, we expected the electric field value of 46 mV/m, and we measured an electric field of 36 mV/m (Figs. S7 and S8). In the effective electrical stimulation results of the in vivo study, the electric fields with intensities ranging from 10 to 338 mV/m [22,63–65]. The strengths of the acoustic (0.3 MPa) and electric fields (36 mV/m) produced in our study are both in the range of the effective stimulus parameters. The electric fields of TMAS are subthreshold stimulation, and some studies have suggested that subthreshold electrical stimulation is a key mechanism driving synaptic plasticity in the brain [66,67]. We used a pulse repetition frequency (PRF) of 1 kHz and an interval of the pulse train of 200 ms, so the repetition frequency of the corresponding train was 5 Hz. The repeated implementation of the train generates an efficient electric neurostimulation, which corresponds to low-frequency stimulation (5 Hz). Studies have found that rTMS delivered at 5 Hz to the prefrontal cortex is used to treat major depressive disorder, and subthreshold 5-Hz rTMS was found to increase motor cortex excitability in healthy humans [67,68]. The TMAS-related static magnetic field supplies an extra energy origin relative to TUS and generates an evoked electric field through the magneto-acoustic effect, which is combined with the mechanical forces of TUS; both of those forces play a role in the effect of TMAS. We choose the frequencies commonly used in ultrasound and electrical stimulation. The effect of TMAS is stronger than that of TUS in our study, so we believe that both electricity and ultrasound play a role in TMAS.

In addition, the Piezo1 channel is not only mechanically stimulated but also strongly modulated by voltage, which may be one of the reasons why the overall effect of TMAS was stronger than that of TUS. The role of Piezo1 in different stages of AD may be different, and complete Piezo1 knockout may be embryonic lethal. However, administration of Piezo1 inhibitors may be a practical and promising method for further elucidating the effects of TMAS in mice [14,16]. GsMTx-4 as an antagonist of Piezo1 also selectively inhibits cation-permeable mechanosensitive channels belonging to the transient receptor potential (TRP) channel families [69,70], but we did not investigate the role of other mechanosensitive ion channels that may be implicated in mediating the microglial response to TMAS; whether TMAS acts on other mechanosensitive ion channels will also be explored in the future. In our following study, we will also optimize the inhibition of Piezo1. Transgenic mice with conditional knockout of Piezo1 in the brain are also a feasible option, which is a promising way to further understand the function of TMAS [16]. In addition, Ca^2+^ can rapidly activate downstream pathways after mechanical stimulation, so inhibiting Ca^2+^ signaling in subsequent experiments could be helpful for investigating the mechanism underlying the function of Piezo1 following TMAS. We are the first group to focus specifically on the effect of TMAS on microglial Piezo1 in AD, and we cannot exclude the possibility that TMAS may also exert its beneficial effects on AD through Piezo1 expressed on other types of brain cells (e.g., astrocytes, neurons, and oligodendrocytes).

## Conclusion

Altogether, we demonstrate for the first time the useful function of TMAS, a technique based on TUS and magnetic field, in activating Piezo1 to enhance the microglial autophagy-mediated clearance of Aβ and attenuate AD-associated synaptic plasticity impairment. These findings reveal the significant role of Piezo1 in TMAS and provide new insight into the mechanism by which Piezo1 transforms TMAS-related mechanical and electrical stimuli into biochemical signals. These findings not only promote the application of TMAS for the therapy of neurological and brain aging diseases in the future but also emphasize the potential of Piezo1-mediated mechanotransduction as a novel therapeutic target for AD.

## Materials and Methods

See the Supplementary Materials.

## Data Availability

All data could be acquired from Prof. Z.L. upon request.

## Supplementary Materials

Supplementary Materials Section S1. Figs. S1 to S8. Table S1.

## References

[1] Wang H, Xu X, Pan YC, Yan Y, Hu XY, Chen R, Ravoo BJ, Guo DS, Zhang T. Recognition and removal of amyloid-β by a heteromultivalent macrocyclic coassembly: A potential strategy for the treatment of Alzheimer’s disease. Adv Mater. 2021;33(4):Article 2006483. 10.1002/adma.202006483 33325586

[2] Hansen DV, Hanson JE, Sheng M. Microglia in Alzheimer’s disease. J Cell Biol. 2018;217(2):459–472. 29196460 10.1083/jcb.201709069 PMC5800817

[3] Eimer WA, Vassar R. Neuron loss in the 5XFAD mouse model of Alzheimer’s disease correlates with intraneuronal Aβ_42_ accumulation and Caspase-3 activation. Mol Neurodegener. 2013;8:2. 23316765 10.1186/1750-1326-8-2 PMC3552866

[4] Wang S, Li K, Zhao S, Zhang X, Yang Z, Zhang J, Zhang T. Early-stage dysfunction of hippocampal theta and gamma oscillations and its modulation of neural network in a transgenic 5xFAD mouse model. Neurobiol Aging. 2020;94:121–129. 32619873 10.1016/j.neurobiolaging.2020.05.002

[5] Ivkovic S, Major T, Mitic M, Loncarevic-Vasiljkovic N, Jovic M, Adzic M. Fatty acids as biomodulators of Piezo1 mediated glial mechanosensitivity in Alzheimer’s disease. Life Sci. 2022;297:Article 120470. 35283177 10.1016/j.lfs.2022.120470

[6] Hammond TR, Robinton D, Stevens B. Microglia and the brain: Complementary partners in development and disease. Annu Rev Cell Dev Biol. 2018;34:523–544. 30089221 10.1146/annurev-cellbio-100616-060509

[7] Sarlus H, Heneka MT. Microglia in Alzheimer’s disease. J Clin Invest. 2017;127(9):3240–3249. 28862638 10.1172/JCI90606 PMC5669553

[8] Jantti H, Sitnikova V, Ishchenko Y, Shakirzyanova A, Giudice L, Ugidos IF, Gomez-Budia M, Korvenlaita N, Ohtonen S, Belaya I, et al. Microglial amyloid beta clearance is driven by PIEZO1 channels. J Neuroinflammation. 2022;19(1):147. 35706029 10.1186/s12974-022-02486-y PMC9199162

[9] Melo P, Socodato R, Silveira MS, Neves MAD, Relvas JB, Mendes PI. Mechanical actuators in microglia dynamics and function. Eur J Cell Biol. 2022;101(3):Article 151247. 35691123 10.1016/j.ejcb.2022.151247

[10] Chi S, Cui Y, Wang H, Jiang J, Zhang T, Sun S, Zhou Z, Zhong Y, Xiao B. Astrocytic Piezo1-mediated mechanotransduction determines adult neurogenesis and cognitive functions. Neuron. 2022. 10.1016/j.neuron.2022.07.010 35963237

[11] Saotome K, Murthy SE, Kefauver JM, Whitwam T, Patapoutian A, Ward AB. Structure of the mechanically activated ion channel Piezo1. Nature. 2018;554(7693):481–486. 29261642 10.1038/nature25453 PMC6010196

[12] Velasco-Estevez M, Gadalla KKE, Linan-Barba N, Cobb S, Dev KK, Sheridan GK. Inhibition of Piezo1 attenuates demyelination in the central nervous system. Glia. 2020;68(2):356–375. 31596529 10.1002/glia.23722

[13] Lai A, Cox CD, Chandra Sekar N, Thurgood P, Jaworowski A, Peter K, Baratchi S. Mechanosensing by Piezo1 and its implications for physiology and various pathologies. Biol Rev Camb Philos Soc. 2022;97(2):604–614. 34781417 10.1111/brv.12814

[14] Velasco-Estevez M, Mampay M, Boutin H, Chaney A, Warn P, Sharp A, Burgess E, Moeendarbary E, Dev KK, Sheridan GK. Infection augments expression of mechanosensing Piezo1 channels in amyloid plaque-reactive astrocytes. Front Aging Neurosci. 2018;10:332. 30405400 10.3389/fnagi.2018.00332 PMC6204357

[15] Zhang G, Li X, Wu L, Qin YX. Piezo1 channel activation in response to mechanobiological acoustic radiation force in osteoblastic cells. Bone Res. 2021;9(1):16. 33692342 10.1038/s41413-020-00124-y PMC7946898

[16] Qiu Z, Guo J, Kala S, Zhu J, Xian Q, Qiu W, Li G, Zhu T, Meng L, Zhang R, et al. The mechanosensitive ion channel Piezo1 significantly mediates in vitro ultrasonic stimulation of neurons. iScience. 2019;21:448–457. 31707258 10.1016/j.isci.2019.10.037 PMC6849147

[17] Singh A, Tijore A, Margadant F, Simpson C, Chitkara D, Low BC, Sheetz M. Enhanced tumor cell killing by ultrasound after microtubule depolymerization. Bioeng Transl Med. 2021;6(3):Article e10233. 34589605 10.1002/btm2.10233 PMC8459596

[18] Menardi A, Rossi S, Koch G, Hampel H, Vergallo A, Nitsche MA, Stern Y, Borroni B, Cappa SF, Cotelli M, et al. Toward noninvasive brain stimulation 2.0 in Alzheimer’s disease. Ageing Res Rev. 2022;75:Article 101555. 34973457 10.1016/j.arr.2021.101555 PMC8858588

[19] Rajasethupathy P, Ferenczi E, Deisseroth K. Targeting neural circuits. Cell. 2016;165(3):524–534. 27104976 10.1016/j.cell.2016.03.047 PMC5296409

[20] Lipsman N, Meng Y, Bethune AJ, Huang Y, Lam B, Masellis M, Herrmann N, Heyn C, Aubert I, Boutet A, et al. Blood-brain barrier opening in Alzheimer’s disease using MR-guided focused ultrasound. Nat Commun. 2018;9(1):2336. 30046032 10.1038/s41467-018-04529-6 PMC6060168

[21] Hu X, Li F, Zeng J, Zhou Z, Wang Z, Chen J, Cao D, Hong Y, Huang L, Chen Y, et al. Noninvasive low-frequency pulsed focused ultrasound therapy for rheumatoid arthritis in mice. Research. 2022;2022. 10.34133/research.0013 PMC11407525 39290964

[22] Zhou X, Liu S, Wang Y, Yin T, Yang Z, Liu Z. High-resolution transcranial electrical simulation for living mice based on magneto-acoustic effect. Front Neurosci. 2019;13:1342. 31920507 10.3389/fnins.2019.01342 PMC6923685

[23] Wang Y, Feng L, Liu S, Zhou X, Yin T, Liu Z, Yang Z. Transcranial magneto-acoustic stimulation improves neuroplasticity in hippocampus of Parkinson’s disease model mice. Neurotherapeutics. 2019;16(4):1210–1224. 30993592 10.1007/s13311-019-00732-5 PMC6985386

[24] Bystritsky A, Korb AS, Douglas PK, Cohen MS, Melega WP, Mulgaonkar AP, DeSalles A, Min BK, Yoo SS. A review of low-intensity focused ultrasound pulsation. Brain Stimul. 2011;4(3):125–136. 21777872 10.1016/j.brs.2011.03.007

[25] Wijerathne TD, Ozkan AD, Lacroix JJ. Yoda1’s energetic footprint on Piezo1 channels and its modulation by voltage and temperature. Proc Natl Acad Sci USA. 2022;119(29):Article e2202269119. 35858335 10.1073/pnas.2202269119 PMC9303978

[26] Moroni M, Servin-Vences MR, Fleischer R, Sanchez-Carranza O, Lewin GR. Voltage gating of mechanosensitive PIEZO channels. Nat Commun. 2018;9(1):1096. 29545531 10.1038/s41467-018-03502-7 PMC5854696

[27] Dinel AL, Lucas C, Guillemet D, Laye S, Pallet V, Joffre C. Chronic supplementation with a mix of *Salvia officinalis* and *Salvia lavandulaefolia* improves Morris water maze learning in normal adult C57Bl/6J mice. Nutrients. 2020;12(6):1777. 32549250 10.3390/nu12061777 PMC7353372

[28] Knobloch M, Mansuy IM. Dendritic spine loss and synaptic alterations in Alzheimer’s disease. Mol Neurobiol. 2008;37(1):73–82. 18438727 10.1007/s12035-008-8018-z

[29] Harry GJ. Microglia during development and aging. Pharmacol Ther. 2013;139(3):313–326. 23644076 10.1016/j.pharmthera.2013.04.013 PMC3737416

[30] Moshayedi P, Ng G, Kwok JC, Yeo GS, Bryant CE, Fawcett JW, Franze K, Guck J. The relationship between glial cell mechanosensitivity and foreign body reactions in the central nervous system. Biomaterials. 2014;35(13):3919–3925. 24529901 10.1016/j.biomaterials.2014.01.038

[31] Condello C, Yuan P, Schain A, Grutzendler J. Microglia constitute a barrier that prevents neurotoxic protofibrillar Aβ_42_ hotspots around plaques. Nat Commun. 2015;6:6176. 25630253 10.1038/ncomms7176 PMC4311408

[32] Lu J, Zhang C, Lv J, Zhu X, Jiang X, Lu W, Lu Y, Tang Z, Wang J, Shen X. Antiallergic drug desloratadine as a selective antagonist of 5HT(2A) receptor ameliorates pathology of Alzheimer’s disease model mice by improving microglial dysfunction. Aging Cell. 2021;20(1):Article e13286. 33369003 10.1111/acel.13286 PMC7811850

[33] Pathak MM, Nourse JL, Tran T, Hwe J, Arulmoli J, Le DT, Bernardis E, Flanagan LA, Tombola F. Stretch-activated ion channel Piezo1 directs lineage choice in human neural stem cells. Proc Natl Acad Sci USA. 2014;111(45):16148–16153. 25349416 10.1073/pnas.1409802111 PMC4234578

[34] Chakraborty M, Chu K, Shrestha A, Revelo XS, Zhang X, Gold MJ, Khan S, Lee M, Huang C, Akbari M, et al. Mechanical stiffness controls dendritic cell metabolism and function. Cell Rep. 2021;34(2):Article 108609. 33440149 10.1016/j.celrep.2020.108609

[35] Liu TT, Du XF, Zhang BB, Zi HX, Yan Y, Yin JA, Hou H, Gu SY, Chen Q, Du JL. Piezo1-mediated Ca^2+^ activities regulate brain vascular pathfinding during development. Neuron. 2020;108(1):180–192.e5. 32827455 10.1016/j.neuron.2020.07.025

[36] Atcha H, Jairaman A, Holt JR, Meli VS, Nagalla RR, Veerasubramanian PK, Brumm KT, Lim HE, Othy S, Cahalan MD, et al. Mechanically activated ion channel Piezo1 modulates macrophage polarization and stiffness sensing. Nat Commun. 2021;12(1):3256. 34059671 10.1038/s41467-021-23482-5 PMC8167181

[37] Kondratskyi A, Kondratska K, Skryma R, Klionsky DJ, Prevarskaya N. Ion channels in the regulation of autophagy. Autophagy. 2018;14(1):3–21. 28980859 10.1080/15548627.2017.1384887 PMC5846505

[38] Yuan J, Liu H, Zhang H, Wang T, Zheng Q, Li Z. Controlled activation of TRPV1 channels on microglia to boost their autophagy for clearance of alpha-synuclein and enhance therapy of Parkinson’s disease. Adv Mater. 2022;34(11):Article e2108435. 35023596 10.1002/adma.202108435

[39] Kirkin V, Rogov VV. A diversity of selective autophagy receptors determines the specificity of the autophagy pathway. Mol Cell. 2019;76(2):268–285. 31585693 10.1016/j.molcel.2019.09.005

[40] Berglund RA-O, Guerreiro-Cacais AA-O, Adzemovic MZ, Zeitelhofer M, Lund HA-O, Ewing EA-OX, Ruhrmann S, Nutma E, Parsa RA-O, Thessen-Hedreul M, et al. Microglial autophagy-associated phagocytosis is essential for recovery from neuroinflammation. Sci Immunol. 2020;5(52):Article eabb5077. 33067381 10.1126/sciimmunol.abb5077

[41] Wyss-Coray T. Inflammation in Alzheimer disease: Driving force, bystander or beneficial response? Nat Med. 2006;12(9):1005–1015. 16960575 10.1038/nm1484

[42] Venegas C, Kumar S, Franklin BS, Dierkes T, Brinkschulte R, Tejera D, Vieira-Saecker A, Schwartz S, Santarelli F, Kummer MP, et al. Microglia-derived ASC specks cross-seed amyloid-β in Alzheimer’s disease. Nature. 2017;552(7685):355–361. 29293211 10.1038/nature25158

[43] Liu S, Liu Y, Hao W, Wolf L, Kiliaan AJ, Penke B, Rube CE, Walter J, Heneka MT, Hartmann T, et al. TLR2 is a primary receptor for Alzheimer’s amyloid β peptide to trigger neuroinflammatory activation. J Immunol. 2012;188(3):1098–1107. 22198949 10.4049/jimmunol.1101121

[44] Yuan Y, Chen Y, Li X. Theoretical analysis of transcranial magneto-acoustical stimulation with Hodgkin-Huxley neuron model. Front Comput Neurosci. 2016;10:35. 27148032 10.3389/fncom.2016.00035 PMC4835452

[45] Geng J, Shi Y, Zhang J, Yang B, Wang P, Yuan W, Zhao H, Li J, Qin F, Hong L, et al. TLR4 signalling via Piezo1 engages and enhances the macrophage mediated host response during bacterial infection. Nat Commun. 2021;12(1):3519. 34112781 10.1038/s41467-021-23683-y PMC8192512

[46] Li L, Li L, Zhou X, Yu Y, Li Z, Zuo D, Wu Y. Silver nanoparticles induce protective autophagy via Ca^2+^/CaMKKβ/AMPK/mTOR pathway in SH-SY5Y cells and rat brains. Nanotoxicology. 2019;13(3):369–391. 30729847 10.1080/17435390.2018.1550226

[47] Velasco-Estevez M, Rolle SO, Mampay M, Dev KK, Sheridan GK. Piezo1 regulates calcium oscillations and cytokine release from astrocytes. Glia. 2020;68(1):145–160. 31433095 10.1002/glia.23709

[48] Eshraghi M, Adlimoghaddam A, Mahmoodzadeh A, Sharifzad F, Yasavoli-Sharahi H, Lorzadeh S, Albensi BC, Ghavami S. Alzheimer’s disease pathogenesis: Role of autophagy and mitophagy focusing in microglia. Int J Mol Sci. 2021;22(7). 10.3390/ijms22073330 PMC8036323 33805142

[49] Bharath LP, Agrawal M, McCambridge G, Nicholas DA, Hasturk H, Liu J, Jiang K, Liu R, Guo Z, Deeney J, et al. Metformin enhances autophagy and normalizes mitochondrial function to alleviate aging-associated inflammation. Cell Metab. 2020;32(1):44–55.e6. 32402267 10.1016/j.cmet.2020.04.015 PMC7217133

[50] Koser DE, Thompson AJ, Foster SK, Dwivedy A, Pillai EK, Sheridan GK, Svoboda H, Viana M, Costa LD, Guck J, et al. Mechanosensing is critical for axon growth in the developing brain. Nat Neurosci. 2016;19(12):1592–1598. 27643431 10.1038/nn.4394 PMC5531257

[51] Murty DV, Manikandan K, Kumar WS, Ramesh RG, Purokayastha S, Nagendra B, Ml A, Balakrishnan A, Javali M, Rao NP, et al. Stimulus-induced gamma rhythms are weaker in human elderly with mild cognitive impairment and Alzheimer’s disease. eLife. 2021;10:Article e61666. 34099103 10.7554/eLife.61666 PMC8238507

[52] Park M, Hoang GM, Nguyen T, Lee E, Jung HJ, Choe Y, Lee MH, Hwang JY, Kim JG, Kim T. Effects of transcranial ultrasound stimulation pulsed at 40 Hz on Aβ plaques and brain rhythms in 5×FAD mice. Transl Neurodegener. 2021;10(1):48. 34872618 10.1186/s40035-021-00274-x PMC8650290

[53] Iaccarino HF, Singer AC, Martorell AJ, Rudenko A, Gao F, Gillingham TZ, Mathys H, Seo J, Kritskiy O, Abdurrob F, et al. Gamma frequency entrainment attenuates amyloid load and modifies microglia. Nature. 2016;540(7632):230–235. 27929004 10.1038/nature20587 PMC5656389

[54] Benussi A, Cantoni V, Grassi M, Brechet L, Michel CM, Datta A, Thomas C, Gazzina S, Cotelli MS, Bianchi M, et al. Increasing brain gamma activity improves episodic memory and restores cholinergic dysfunction in Alzheimer’s disease. Ann Neurol. 2022;92(2):322–334. 35607946 10.1002/ana.26411 PMC9546168

[55] Liu Y, Wang G, Cao C, Zhang G, Tanzi EB, Zhang Y, Zhou W, Li Y. Neuromodulation effect of very low intensity transcranial ultrasound stimulation on multiple nuclei in rat brain. Front Aging Neurosci. 2021;13:Article 656430. 33935688 10.3389/fnagi.2021.656430 PMC8081960

[56] Xiang S, Zhang Y, Jiang T, Ke Z, Shang Y, Ning W, Yang Z, Zhang T. Knockdown of Follistatin-like 1 disrupts synaptic transmission in hippocampus and leads to cognitive impairments. Exp Neurol. 2020;333:Article 113412. 32721453 10.1016/j.expneurol.2020.113412

[57] Kisler K, Nelson AR, Montagne A, Zlokovic BV. Cerebral blood flow regulation and neurovascular dysfunction in Alzheimer disease. Nat Rev Neurosci. 2017;18(7):419–434. 28515434 10.1038/nrn.2017.48 PMC5759779

[58] Perez-Neri I, Gonzalez-Aguilar A, Sandoval H, Pineda C, Rios C. Therapeutic potential of ultrasound neuromodulation in decreasing neuropathic pain: Clinical and experimental evidence. Curr Neuropharmacol. 2021;19(3):334–348. 32691714 10.2174/1570159X18666200720175253 PMC8033967

[59] Kubanek J. Neuromodulation with transcranial focused ultrasound. Neurosurg Focus. 2018;44(2):E14. 10.3171/2017.11.FOCUS17621 PMC5927579 29385924

[60] Qiu Z, Kala S, Guo J, Xian Q, Zhu J, Zhu T, Hou X, Wong KF, Yang M, Wang H, et al. Targeted neurostimulation in mouse brains with non-invasive ultrasound. Cell Rep. 2020;32(7):Article 108033. 32814040 10.1016/j.celrep.2020.108033

[61] Lin Z, Meng L, Zou J, Zhou W, Huang X, Xue S, Bian T, Yuan T, Niu L, Guo Y, et al. Non-invasive ultrasonic neuromodulation of neuronal excitability for treatment of epilepsy. Theranostics. 2020;10(12):5514–5526. 32373225 10.7150/thno.40520 PMC7196311

[62] Kim T, Park C, Chhatbar PY, Feld J, Mac Grory B, Nam CS, Wang P, Chen M, Jiang X, Feng W. Effect of low intensity transcranial ultrasound stimulation on neuromodulation in animals and humans: An updated systematic review. Front Neurosci. 2021;15:Article 620863. 10.3389/fnins.2021.620863 PMC8079725 33935626

[63] Turner DA, Degan S, Galeffi F, Schmidt S, Peterchev AV. Rapid, dose-dependent enhancement of cerebral blood flow by transcranial AC stimulation in mouse. Brain Stimul. 2021;14(1):80–87. 33217607 10.1016/j.brs.2020.11.012 PMC7855527

[64] Van Hoornweder S, Caulfield KA, Nitsche M, Thielscher A, Meesen RLJ. Addressing transcranial electrical stimulation variability through prospective individualized dosing of electric field strength in 300 participants across two samples: The 2-SPED approach. J Neural Eng. 2022;19(5):Article 056045. 10.1088/1741-2552/ac9a78 PMC9855635 36240729

[65] Louviot S, Tyvaert L, Maillard LG, Colnat-Coulbois S, Dmochowski J, Koessler L. Transcranial electrical stimulation generates electric fields in deep human brain structures. Brain Stimul. 2022;15(1):1–12. 34742994 10.1016/j.brs.2021.11.001

[66] Tang AD, Bennett W, Bindoff AD, Bolland S, Collins J, Langley RC, Garry MI, Summers JJ, Hinder MR, Rodger J, et al. Subthreshold repetitive transcranial magnetic stimulation drives structural synaptic plasticity in the young and aged motor cortex. Brain Stimul. 2021;14(6):1498–1507. 34653682 10.1016/j.brs.2021.10.001

[67] Jiang W, Isenhart R, Sutherland R, Lu Z, Xu H, Pace J, Bonaguidi MA, Lee DJ, Liu CY, Song D. Subthreshold repetitive transcranial magnetic stimulation suppresses ketamine-induced poly population spikes in rat sensorimotor cortex. Front Neurosci. 2022;16:Article 998704. 36340783 10.3389/fnins.2022.998704 PMC9633989

[68] Tang ZM, Xuan CY, Li X, Dou ZL, Lan YJ, Wen HM. Effect of different pulse numbers of transcranial magnetic stimulation on motor cortex excitability: Single-blind, randomized cross-over design. CNS Neurosci Ther. 2019;25(11):1277–1281. 31696644 10.1111/cns.13248 PMC6834918

[69] Gnanasambandam R, Ghatak C, Yasmann A, Nishizawa K, Sachs F, Ladokhin AS, Sukharev SI, Suchyna TM. GsMTx4: Mechanism of inhibiting mechanosensitive ion channels. Biophys J. 2017;112(1):31–45. 28076814 10.1016/j.bpj.2016.11.013 PMC5231890

[70] Romac JM, Shahid RA, Swain SM, Vigna SR, Liddle RA. Piezo1 is a mechanically activated ion channel and mediates pressure induced pancreatitis. Nat Commun. 2018;9(1):1715. 29712913 10.1038/s41467-018-04194-9 PMC5928090

[71] Paxinos G, Franklin KB. The mouse brain in stereotaxic coordinates (2nd ed). 2nd ed. USA: Elsevier; 2004.

