# Supplementary material for: Transcranial Magneto-Acoustic Stimulation Attenuates Synaptic Plasticity Impairment through the Activation of Piezo1 in Alzheimer’s Disease Mouse Model
Source: Research (Wash D C). 2023 May 8;6:0130. doi: 10.34133/research.0130 (PMC10202414; doi:10.34133/research.0130)
Supplement: Supplementary Materials — Section S1. Figs. S1 to S8. Table S1. [file research.0130.f1.zip › Revised Supplementary Materials (Clean Version).docx]

Transcranial Magneto-Acoustic Stimulation Attenuates Synaptic Plasticity Impairment Through the Activation of Piezo1 in Alzheimer's Disease Mouse Model

Fangxuan Chu^1^, Ruxin Tan^1^, Xin Wang^1^, Xiaoqing Zhou^1^, Ren Ma^1, 3^, Xiaoxu Ma^1^, Ying Li^1^, Ruixu Liu^1^, Chunlan Zhang^1^, Xu Liu^1^, Tao Yin^1,2^*, Zhipeng Liu^1^*

*Corresponding Author：

Tao Yin, PhD

^1^Institute of Biomedical Engineering, Chinese Academy of Medical Sciences and Peking Union Medical College, Tianjin, 300192, China.

^2^Neuroscience Center, Chinese Academy of Medical Science & Peking Union Medical College, Beijing, 100730, China

^3^Tianjin Institutes of Health Science, Tianjin, 301600, China.

Email address: [bme500@163.com](mailto:bme500@163.com)

Phone numbers: +8613802093408

Zhipeng Liu, PhD

^1^Institute of Biomedical Engineering, Chinese Academy of Medical Sciences and Peking Union Medical College, Tianjin, 300192, China.

Email address: [lzpeng67@163.com](mailto:lzpeng67@163.com)

Phone numbers: +861301130189

**Supplementary Materials**

# Materials and methods

### Materials

**Table S1.** Key resources of experiment.

| Reagent | Source | Identifier |
| --- | --- | --- |
| anti-β-amyloid | CST | 14974 |
| anti-β-amyloid | Biolegend | 805501 |
| anti-SYP (synaptophysin) | Abcam | ab32127 |
| anti-PSD-95 (postsynaptic density protein 95) | Abcam | ab238135 |
| anti-NR-2B (NMDAR2B) | Abcam | ab65783 |
| anti-NR-2A NMDAR2A | Abcam | ab124913 |
| anti-DBN (Drebrin) | EnzoBiochem | ADI-NBA-110 |
| anti-Iba1 | Wako Chemicals | 019-19741 |
| anti-Iba1 | Wako Chemicals | 016-26721 |
| anti-NeuN | CST | 24307 |
| anti-Piezo1 | Novus Biologicals | NBP2-75617 |
| anti-CD86 | Abcam | ab213044 |
| anti-CD206 | Abcam | ab8918 |
| anti-LC3 | CST | 83506 |
| anti-CD68 | Abcam | ab53444 |
| anti-iNOS | CST | 13120S |
| anti-COX2 | CST | 12282S |
| anti-IL-1β | Abcam | ab254360 |
| anti-IL-6 | CST | 12912S |
| anti-TNF-α | CST | 11948S |
| anti-IL-10 | Abcam | ab189392 |
| anti-IL-4 | Thermo Fisher | PA5-25165 |
| anti-p-CAMKII | Abcam | ab171095 |
| anti-CAMKII | Abcam | ab134041 |
| anti-p-AMPK | CST | 2535T |
| anti- AMPK | CST | 5831S |
| anti-p-mTOR | CST | 5536T |
| anti-mTOR | CST | 2983T |
| anti-Beclin-1 | CST | 3738S |
| anti-p62 | CST | 5114S |
| anti- LC3A/B | CST | 4108 |
| anti-GAPDH | Abcam | ab8245 |
| Goat anti-mouse IgG (H+L) Alexa Fluor 488 conjugate | Thermo Fisher Scientific | A-11029 |
| Goat anti-rabbit IgG (H+L) Alexa Fluor 647 conjugate | Thermo Fisher Scientific | A-27040 |
| Human Amyloid β Oligomers (82E1-specific) Assay Kit | Immuno-Biological Laboratories Co., Ltd | #27725 |
| Human Amyloid beta (aa1-42) Quantikine ELISA Kit | R&D | DAB142 |
| PrimeScript RT Master Mix | Vazyme | R323 |
| SYBR Green I Mixture | Vazyme | Q712 |
| HiLyte FluorTM 555-labeled Aβ | AnaSpec, Inc | AS-60480-01 |
| Amyloid β Protein Fragment 1-42 | Macklin | A834109 |

### Animals

5xFAD transgenic mice and their wild-type littermates were purchased from Beijing HFK BioTechnology Co. Ltd. (Beijing, China). The 5xFAD mice overexpressing human APP and PSEN1 proteins under the control of the Thy1.2 promoter, with five AD-linked mutations: the Swedish (K670N/M671L), Florida (I716V) and London (V717I) mutations in APP, and the M146L and L286V mutations in PSEN1. The accumulation of five FAD mutations mainly affects Aβ production, which can express a large number of Aβ42. All mice were maintained on a 12 h light/dark cycle at a suitable temperature (23–25 °C) and humidity (50–60%) at the Chinese Academy of Medical Sciences & Peking Union Medical College. All mice had free access to adequate food and water. All animal experimental procedures were conducted in accordance with protocols approved by the Animal Care and Ethics Committee of the Chinese Academy of Medical Sciences & Peking Union Medical College, and every effort was made to alleviate the suffering of the animals and reduce the number of animals used.

### Experimental design

The experimental design is presented in Fig. 1a. The mice (half male and half female) were randomly divided into the WT + Sham group (n = 18), AD + Sham group (n = 18), AD + TUS group (n = 16), AD + TMAS group (n = 18), AD + TUS + GsM group (n = 18) and AD + TMAS + GsM group (n = 18). The AD + TUS + GsM and AD + TMAS + GsM groups were first injected with the Piezo1 antagonist GsMTx-4 (20 µM, 1ul, ab141871) into the bilateral lateral ventricles (stereotaxic coordinates: 0.5 mm posterior to bregma, 1.1 mm lateral to the midline, and 2.5 mm ventral to bregma) using a microsyringe (Hamilton, Switzerland) at a rate of 0.5 µl/min. The microsyringe was kept in place for 10 min after injection to allow for diffusion of the drug and then slowly retracted (Fig. 1b). After one week of recovery, the mice in the AD + TUS and AD + TUS + GsM groups received TUS of the bilateral hippocampus for 5 min daily for 28 days, and the mice in the AD + TMAS and AD + TMAS + GsM groups received TMAS (see Section 2.4) of the bilateral hippocampus for 5 min daily for 28 days. Half of the mice in each group were randomly selected for cerebral blood flow (CBF) monitoring, novel object recognition (NOR) test, Y maze test and Morris water maze (MWM) test. After the behavioral experiment, mice were sacrificed and brains were removed for RNA sequencing (RNA-seq). Twenty-four hours after the last stimulation, the other half of the mice were performed the standard procedure for *in vivo* electrophysiological recordings. After the electrophysiological experiment, mice were sacrificed and brains were removed for ELISA, immunostaining, Western blotting, Golgi-Cox staining and quantitative real-time PCR (RT‒qPCR).

### TMAS and TUS treatment

We designed complete TUS and TMAS systems (Fig. 1c). The TMAS system consisted of 2 function generators (TFG6920A, Shuying, China; AFG3252, Tektronix, United States), an RF amplifier (GA2500, RITEC, United States), a 1 MHz focused US transducer (FP-1M, IOA-AC, China; Supplemental Fig. S7), an oscilloscope (MSO4104, Tektronix, United States) and a calibrated hydrophone (NH-1000, British PA company). A static magnetic field for TMAS was generated by a permanent magnet, and the strength was tested via a Gauss meter (Model 475, Lakeshore, United States), the magnetic field strength at the stimulation target was about 0. 2 T. The stimulation parameters for TMAS and TUS were as follows (Fig. 1d): stimulus consisting of 200 tone burst pulses, 1MHz center frequency, 40% duty cycle, 1 kHz pulse repetition frequency, peak positive acoustic pressure of 0.3 MPa, duration of 5 min. According to the measured peak-to-peak value of the voltage signal (255mV, Supplemental Fig. S8) and the sensitivity (850mV/ MPa) of hydrophone, the peak-to-peak value of acoustic pressure used was 0.3 MPa in our study. TMAS was delivered to the bilateral hippocampus (2.0 mm anteroposterior, ± 1.4 mm mediolateral, and 1.5 mm dorsoventral from bregma)^[1]^ and TUS was delivered to the same brain region in the absence of a static magnetic field. One transducer was used to sequentially stimulate each side of the hippocampus. During the stimulation, the mice were anesthetized with 1% isoflurane and placed on a stereotaxic instrument. Mice in the WT + Sham and AD + Sham groups were anesthetized with isoflurane and subjected to sham stimulation by turning off the stimulated signal that with equally static magnetic field and the equally conditions as the TMAS and TUS groups.

### Behavioral tests

The behavioral tests were executed in a dark room from 8:00 to 20:00. The experimental apparatus was cleaned with 70% ethanol before and after each trial to eliminate any odor cues. All behavioral experiments were conducted after treatment by a blinded observer.

### Novel object recognition (NOR) test

The novel object recognition (NOR) test was executed as previously described with a 35 × 35 × 25 cm open-field apparatus^[2]^. During the habituation phase, the mice were placed in a box and allowed to explore freely for 5 min for adaptation to the environment. After 24 h, the training phase began. The mice were placed in a box containing two similar objects and allowed to explore freely for 5 min. After 2 h, test 1 was performed. One of the two objects was replaced with a new object and the mice were allowed to explore the two objects for 5 min. After 24 h, test 2 was conducted. The novel object was replaced with another new object, and the same experimental procedures were performed as in test 1. The recognition index (RI) was calculated by the equation: Time_novel_/(Time_novel_ + Time_old_)*100% and Visits_novel_/(Visits_novel_ + Visits_old_)*100%.

### Y maze test

The Y maze test was performed to assess the short-term spatial working memory of the mice after treatment^[3]^. The Y maze was a horizontal maze consisting of three arms (35 × 8 × 18 cm). The mice were placed at the end of one arm of the Y maze and allowed to freely explore the maze for 10 min. The total number of arms entered and the sequence of arms entered were recorded. The spontaneous alternation percentage was calculated using the following equation: (sequential entries into the three different arms/ total number of arms entered-2) × 100%.

### Morris water maze (MWM) test

The Morris water maze (MWM) test was performed to examine the abilities of spatial learning and memory after treatment^[4]^. The MWM test was conducted in a circular pool (100 cm in diameter and 70 cm high), filled with water that was dyed white by nontoxic TiO2. The water temperature was maintained at 23-25 °C, and the depth of water was 40 cm. The swimming activities of the mice were monitored by a CCD camera connected to a computer, through which the data were acquired and analyzed. The MWM test consisted of the initial training (IT), spatial exploration test (SET), reversal training (RT) and reversal exploration test (RET) phases. The swimming pool was equally divided into 4 quadrants (I–IV), and in the IT phase, a platform (diameter of 8 cm) was placed 1–2 cm below the water surface in quadrant I. The mice were placed in the water from the four different quadrants (Ⅰ-Ⅳ) and subjected to training trials for 5 consecutive days (4 trials per day), during which they were allowed to search the hidden platform for 60 s. If a mouse found the platform, it was allowed to stay on it for 3 s, if not, it was guided to the platform and required to stay on it for at least 10 s. The time spent on finding the platform (escape latency), swimming speed and swimming path in each trial were recorded. Cognitive performance was scored according to the following scale, with a higher score indicating a more effective cognitive strategy: thigmotaxis = 1, random = 2, corrected = 3 and direct = 4. The cognitive score was calculated as = (the total score in the 4 trials/16) ×100%. During the SET phase, the platform was removed from quadrant I, and the mice were released into the pool from the platform opposite the one that previously contained the platform (quadrant III) and allowed to swim for 60 s. The spent time in the target quadrant and the number of platform crossings were recorded. In the RT phase, the learning flexibility of the mice was tested by placing the platform in the middle of quadrant III. The mice were subjected to training trials for 3 consecutive days (four trials per day) during the RT phase, and the RET was performed 24 h after the last trial. The experimental procedures were similar to those of the IT phase, and the swimming speed, swimming path, number of platform crossings and escape latency were recorded.

### **In vivo** **electrophysiological recordings**

*In vivo* electrophysiological recordings were performed to record the synaptic responses from the perforant pathway (PP) to the dentate gyrus (DG)^[5]^. Briefly, mice were anesthetized with 30 % urethane (0.4 mL/kg, i.p.) and then located using a stereotaxic frame (SN-3, Narishige, Japan). Body temperature was maintained with a heating pad. Stimulating and recording electrodes were stereotaxically implanted in the PP (anteroposterior:3.8, mediolateral:3, dorsoventral:1.5) and DG (anteroposterior:2.0, mediolateral:1.4, dorsoventral:1.5) of the hippocampus through a hole drilled in the skull. First, the response of the PP to a single-pulse stimulus with a strength (range 0.3–0.5 mA, stimulus pulse with 0.2 ms, at 0.03 Hz) that induced 70% of the maximum response was recorded every 30 s for 30 min as the baseline response. Then, local field potentials (LFPs) were obtained at a sampling rate of 1000 Hz for 15 min. After that, theta burst stimulation (TBS, 30 trains of 12 pulses, 200 Hz at 5 Hz) was applied to trigger LTP, which was recorded every 60 s for 1 h. Finally, low-frequency stimulation (LFS; 1 Hz, 15 min) was used to trigger DEP, which was recorded every 60 s for 1 h. All initial data analysis was performed with Clampfit 10.0 software (Molecular Devices, Sunnyvale, CA, USA). The slope of the field excitatory postsynaptic potential (fEPSP) was used to evaluate synaptic efficacy.

### Power spectrum density (PSD) analysis

The LFP data were obtained at a sampling rate of 1000 Hz for 20 minutes and were analyzed offline using built-in and custom- written MATLAB codes (Mathworks). A multi-window spectrum calculation method is used to measure the PSD with a data window length of 10000 (10s) and an overlap rate of 50%. The total power in the range of 0.5-100Hz band, the relative power of theta band (3-8Hz), lowgamma band (30-50Hz) and highgamma band (50-100Hz) was standardized to relative percentages across the total power.

### N:M phase-phase coupling analysis

The n:m phase-phase coupling is used to assess the synchronization among different rhythms, the ratio n:m indicates n periods of high-frequency oscillations stabilized for every m periods of low-frequency oscillations. The radial distance, r value, is calculated using the following equation:

$$r_{n:m}=|\frac{1}{N}\sum_{j=1}^{N} exp\left( i \left[ {m\varphi}_{low}\left( j \right)-{n\varphi}_{high}\left( j \right) \right] \right)|$$

### Phase-amplitude coupling (PAC)

The modulation index (MI) is used to determine the intensity of the phase-amplitude coupling between theta and gamma rhythms. The phase of the low-frequency rhythm and the amplitude of the high-frequency rhythm are obtained by the Hilbert transform. Each period of the phase is separated into n intervals, and the mean of the amplitudes within each interval is measured. The probability distribution of the amplitude of the high-frequency oscillations in the low-frequency phase is obtained by normalizing each mean value by the combination of all interval amplitudes. a larger value of MI represents a stronger PAC.

### **Golgi-Cox staining**

Golgi staining was used to assess the dynamic changes in the dendritic spines of neurons in the hippocampal DG and CA1 region^[6]^. Dissected brain tissue was immersed in Golgi-Cox solution at room temperature away from light for 14 days. The brain tissues were cut into 150 μm thick coronal slices using a vibrating microtome (VT1000S, Leica, Germany). The slices were incubated with: 6% Na_2_CO_3_ for 20 min, 70% EtOH for 10 min, 90% EtOH for 15 min, 100% EtOH for 20 min and xylene for 20 min. The brain slices were mounted on a slide with neutral resin and cover slipped. The morphology of dendritic spines in the DG and CA1 regions was assessed using a light microscope (DM3000, Leica, Germany), and the density of dendritic spines was measured using ImageJ software. To calculate the number of dendritic spines, straight branches with a clear resolution and a length exceeding 10 μm were counted. In addition, we classified the dendritic spines into four types according to their morphology: filopodia (long spines with no head), thin (long spines with a small head and a diameter not longer than 0.6 µm), mushroom (long spines with a large head and a diameter longer than 0.6 µm) and stubby (short spines with no head)^[7]^.

### **Immunofluorescence and Immunohistochemistry**

The mice were slowly perfused with phosphate-buffered saline (PBS, pH= 7.4) followed by 4% paraformaldehyde (PFA) under anesthesia, and the brains of the mice were fixed in 4% PFA for 48 h. Brains tissues containing the hippocampus were cut into 35 μm coronal sections using a vibratome (VT1000S, Leica, Germany) for immunofluorescence staining. The brain sections were washed in PBS for 3 × 5 min and permeabilized in 0.5% Triton X-100 at room temperature for 10 min. The sections were washed with PBS (3 × 5 min) and blocked with 10% normal goat serum (NGS) at room temperature for 1 h. After that, the sections were incubated with primary antibodies (1:500) overnight at 4℃. On the second day, the brain sections were washed in PBS for 3 × 5 min and incubated with Alexa 488 (1:1000; Thermo Fisher Scientific, A-11029) or Alexa 647 (1:500; Thermo Fisher Scientific, A-27040) conjugated secondary antibodies for 1 h at room temperature. The nuclei were stained with DAPI for 5 min and imaged under a fluorescence microscope (Olympus, FV1000, Japan). The fluorescence intensity was quantified in ImageJ software.

For immunohistochemical staining, signals were detected by biotinylated IgG, and then the tissues were incubated with streptavidin-conjugated horseradish peroxidase (Vectastain ABC Kit, China), and reacted with hydrogen peroxide (DAB Kit, China). Images were acquired by a Leica microscope. The number of Iba1-positive cells was calculated using ImageJ software.

### **Cells culture and treatment**

BV2 cells are derived from immortalized mouse microglia, and retain the morphological and functional characteristics of microglia. BV2 microglia were cultured in DMEM containing 10% FBS and 1% penicillin/streptomycin in 5% CO2 at 37 °C. The medium was changed every 2 days, and the cells were passaged 2 or 3 days. For TMAS and TUS treatment, cells were seeded in 35 mm sterile cell culture dishes at a density of 1 x 10^6^ cells per dish and treated with TMAS and TUS after cell attachment. The cells were treated with HiLyte FluorTM 555-labeled Aβ at a concentration of 500 nM (AS-60480-01, AnaSpec, Inc) for 12 h and divided into the Aβ-treated group (Aβ) group, TUS-treated (TUS + Aβ) group and TMAS-treated (TMAS + Aβ) group according to the treatments administered. Cells in the TUS + Aβ + GsM and TMAS + Aβ + GsM groups were pretreated with 20 µM GsMTx-4 for 30 min in an incubator, and then subjected to TUS and TMAS treatment, respectively. The cells were then used for subsequent experimental analysis.

### **Transwell migration assay**

Cells were plated in the upper chamber of a Costar Transwell system at a density of 1 × 10^4^ cells/well, and cultured for 12 h at 37 °C in an incubator. Briefly, the cells in each group were fixed with 4% PFA for 20 min, permeabilized with methanol for 10 min and stained with 0.5% crystal violet for 10 min. Finally, the nonmigrated cells were removed with a cotton swab. Images were obtained using a Leica microscope (Wetzlar, Germany), and the number of cells was determined with ImageJ software.

### **Enzyme-linked immunosorbent assay (ELISA)**

Hippocampal and cortical tissues were collected, homogenized and centrifuged at 12000g at 4 °C for 15 min. The supernatant was collected and prepared for subsequent experimental analysis. The levels of Aβ_1-42_ and Aβ_1-42_ oligomers in the hippocampus and cortex were measured following the manufacturer’s instructions by using a human Aβ (aa1-42) ELISA kit (R&D, DAB142) or human Aβ oligomer assay kit (Immuno-Biological Laboratories Co., Ltd. #27725).

### **Western blotting**

Mouse hippocampal and cortical tissues were dissected, lysed in 150 μL RIPA buffer containing 1% PMSF, and incubated on ice for 15 min. The lysates were then centrifuged at 12000 × g for 15 min at 4 °C, and the supernatant was collected. After the protein concentration was quantitated with BCA protein assay kits according to the manufacturer’s instructions (Beyotime Biotechnology, China), the supernatant was mixed with 5× loading buffer and boiled at 95 °C for 10 min. Samples containing equal amounts of protein (30 µg) were separated by 8-12% SDS‒PAGE and transferred to polyvinylidene fluoride (PVDF) membranes (Millipore, USA). The PVDF membranes were then blocked with 5% skim milk for 1 h at room temperature and incubated overnight at 4 °C with primary antibodies (1:1000). The following day, PVDF membranes were washed with TBST for 4 × 10 min and incubated with secondary antibody for 1 h at room temperature. After washing the membranes with TBST for 4 × 10 min, the PVDF membranes were imaged by a chemiluminescent system. The protein band densities were quantified and analyzed in ImageJ software.

### **RT–qPCR**

Total RNA was isolated from the hippocampal and cortical tissues of mice using TRIzol solution (Invitrogen, USA) according to the manufacturer’s instructions, and the RNA concentration was measured using a NanoDrop spectrophotometer (Thermo Fisher Scientific, USA). The RNA was reverse transcribed into cDNA using PrimeScript RT Master Mix (Vazyme, R323). RT–qPCR was conducted with SYBR Green I Mixture (Vazyme, Q712) using the 7500 Fast Real-time PCR System (Applied Biosystems, USA). The primer sequences were as follows: TNFα, 5′-TGCCTATGTCTCAGCCTCTTC-3′ and 5′-GGTCTGGGCCATAGAACTGA-3′; IL-1β, 5′-ATTGTGGCTGTGGAGAAG-3′ and 5′-TTGTGAGGTGCTGATGTA-3′; IL-6, 5′-TGTGCAATGGCAATTCTGAT-3′ and 5′- GGTACTCCAGAAGACCAGAGGA-3′; IL-4, 5′-GGTCTCAACCCCCAGCTAGT-3′ and 5′-GCCGATGATCTCTCTCAAGTGAT-3′; IL-10, 5′-TGTGTCAGCCCTCAGAGTAC-3′ and 5′-CACTGACACTTCGCACAA-3′; IL-13, 5′-CAATTGCAATGCCATCTACAGGAC-3′ and 5′-CGAAACAGTTGCTTTGTGTAGCTGA-3′; Piezo1, 5′-TCATCATCCTTAACCACATGGTG-3′ and 5′-TGAAGACGATAGCTGTCATCCA-3′; GAPDH, 5′- AGGTCGGTGTGAACGGATTTG-3′ and 5′-TGTAGACCATGTAGTTGAGGTCA-3′. mRNA expression levels were normalized to the level of GAPDH using the 2-ΔΔCT method.

### **Cerebral blood flow (CBF) monitoring**

CBF was measured using a laser speckle blood flow imaging system (RFLSI Pro, RWD). CBF was measured on days 0 and 28 of TMAS and TUS treatment. The mice were anesthetized with 1% isoflurane, and fixed with on stereotaxic frame (SN-3, Narishige, Japan), and body temperature was maintained at 35.5-36.5 °C with a heating pad.^[8]^ The skull was exposed by making an incision in the scalp, and imaged color-coded blood flow with a CCD camera located above the head. The main venous blood flow was used as an index for evaluation.

### RNA sequencing (RNA-seq) analysis

RNA was isolated from mouse hippocampal tissues using TRIzol, and RNA integrity was evaluated using the Agilent 2100 bioanalyzer system. High-quality samples were used for library preparation. After passing the library check, the library preparations were sequenced using an Illumina NovaSeq 6000 platform (Illumina, USA), and 150 bp paired-end reads were produced. HISAT2 v2.0.5 was used to construct the index of reference genome and compare the paired end clean reads to the reference genome. Differential expression analysis was performed with DESeq2 software (1.20.0). For analyze differential gene expression, Benjamini and Hochberg's method was used to adjust the P value. Genes with an adjusted P value <= 0.05, which was considered significant, and a |log2FoldChange|>=0.0 according to DESeq2 were considered differentially expressed. Fragments per kilobase million (FPKM) was also used to quantify gene expression levels. Gene Ontology (GO) enrichment analysis was conducted to evaluated the major biological functions of the differentially expressed genes. The differentially expressed genes were subjected to GO enrichment analysis using ClusterProfiler (3.8.1) software. Terms with a P value <= 0.05 were regarded as significantly enriched. We used the GSEA analysis tool (<http://www>. broadinstitute.org/gsea/index.jsp) to perform GSEA of the GO data. Heatmaps, volcano plots, bar plots and bubble plots were generated by the pheatmap, ggplot2, ggrepel and GOplot packages in R.

### Statistical analysis

All data are presented as the means ± SEM. SPSS 26.0 and GraphPad Prism 8 software were used for data analysis and graphing, respectively. The significance of differences was assessed using one-way ANOVA or two-way ANOVA followed by the LSD multiple -comparisons test. When the interaction effect was significant, Bonferroni’s post hoc comparison was performed. The significance level was set at p < 0.05.

# Authors’ contributions

Fangxuan Chu: Writing – original draft, methodology and investigation; Ruxin Tan and Xin Wang: Data curation, Supervision and Software; Xiaoqing Zhou and Ren Ma: Methodology and resources; Xiaoxu Ma: Conceptualization and data curation; Yin Li and Ruxu Liu: Formal analysis and visualization; Chunlan Zhang and Xu Liu: Methodology and validation; Tao Yin and Zhipeng Liu: Writing – review and editing. All authors reviewed and agreed to the current manuscript.

# Financial Support

This work was funded by the National Natural Science Foundation of China (81927806); National Natural Science Foundation of China (52107241); National Natural Science Foundation of China General project (52077223); Chinese Academy of Medical Sciences Medical and Health Science and Technology Innovation Project Mission Statement (2021-I2M-1-058).

# Data Availability

All data and materials could be acquire from Prof. Liu with reasonable request.

# Competing of interests

All the authors declare no conflicts of interest.

### Methods references

1. Qiu Z, Guo J, Kala S, Zhu J, Xian Q, Qiu W, Li G, Zhu T, Meng L, Zhang R *et al*: **The Mechanosensitive Ion Channel Piezo1 Significantly Mediates In Vitro Ultrasonic Stimulation of Neurons**. *iScience* 2019, **21**:448-457.

2. Leger M, Quiedeville A, Bouet V, Haelewyn B, Boulouard M, Schumann-Bard P, Freret T: **Object recognition test in mice**. *Nat Protoc* 2013, **8**(12):2531-2537.

3. Ding B, Lin C, Liu Q, He Y, Ruganzu JB, Jin H, Peng X, Ji S, Ma Y, Yang W: **Tanshinone IIA attenuates neuroinflammation via inhibiting RAGE/NF-kappaB signaling pathway in vivo and in vitro**. *J Neuroinflammation* 2020, **17**(1):302.

4. Bromley-Brits K, Deng Y, Song W: **Morris water maze test for learning and memory deficits in Alzheimer's disease model mice**. *J Vis Exp* 2011(53).

5. Xu X, Xiao X, Yan Y, Zhang T: **Activation of liver X receptors prevents emotional and cognitive dysfunction by suppressing microglial M1-polarization and restoring synaptic plasticity in the hippocampus of mice**. *Brain Behav Immun* 2021, **94**:111-124.

6. Zaqout S, Kaindl AM: **Golgi-Cox Staining Step by Step**. *Front Neuroanat* 2016, **10**:38.

7. Sorra KE, Harris KM: **Overview on the structure, composition, function, development, and plasticity of hippocampal dendritic spines**. (1050-9631 (Print)).

8. Eguchi K, Shindo T, Ito K, Ogata T, Kurosawa R, Kagaya Y, Monma Y, Ichijo S, Kasukabe S, Miyata S *et al*: **Whole-brain low-intensity pulsed ultrasound therapy markedly improves cognitive dysfunctions in mouse models of dementia - Crucial roles of endothelial nitric oxide synthase**. *Brain Stimul* 2018, **11**(5):959-973.
