# Supplementary figures and images for: Transcranial Magneto-Acoustic Stimulation Attenuates Synaptic Plasticity Impairment through the Activation of Piezo1 in Alzheimer’s Disease Mouse Model
Source: Research (Wash D C). 2023 May 8;6:0130. doi: 10.34133/research.0130 (PMC10202414; doi:10.34133/research.0130)

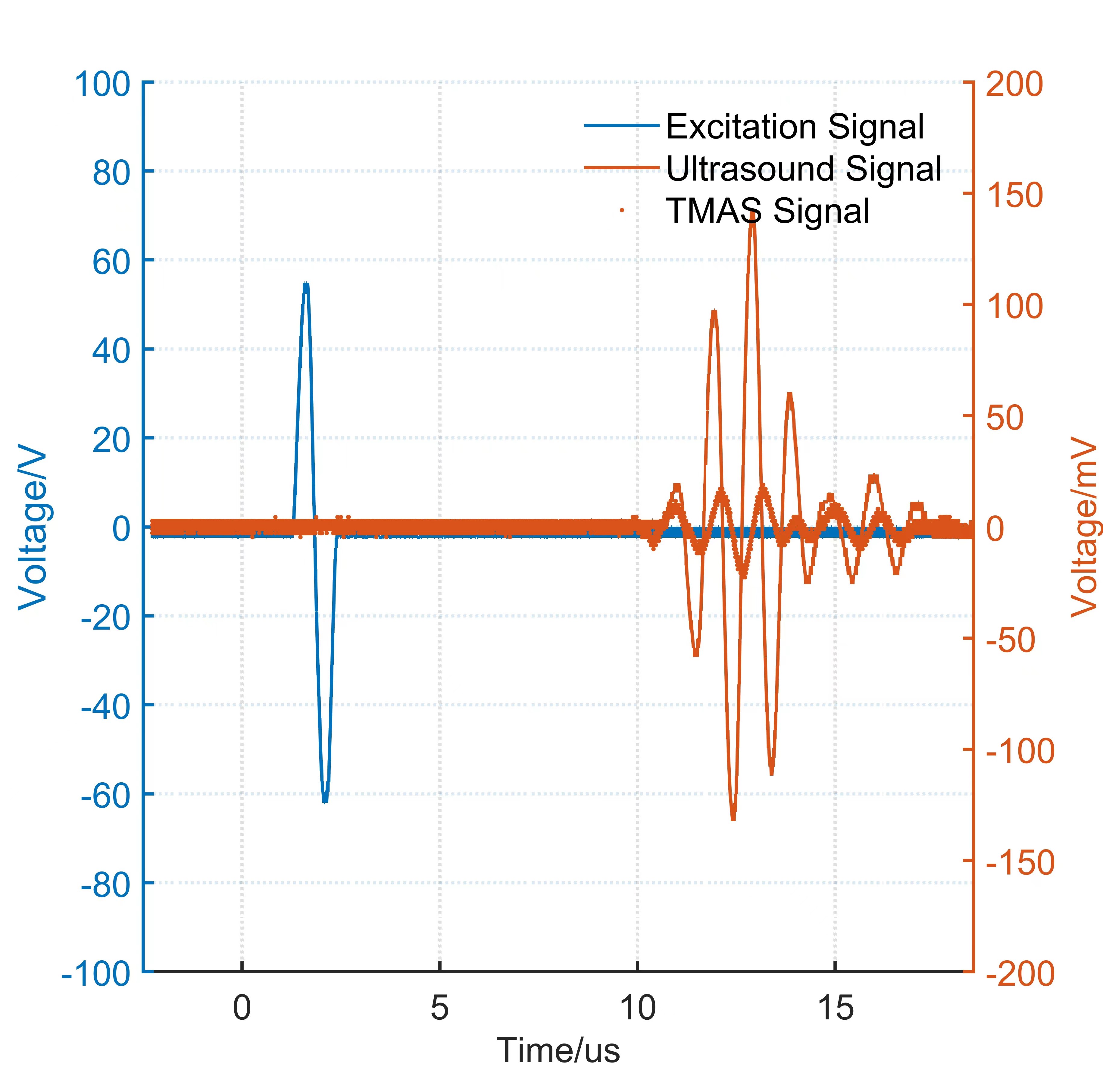

Supplement: Supplementary Materials — Section S1. Figs. S1 to S8. Table S1. [file research.0130.f1.zip › rev-Supplemental Fig. S8.jpg]

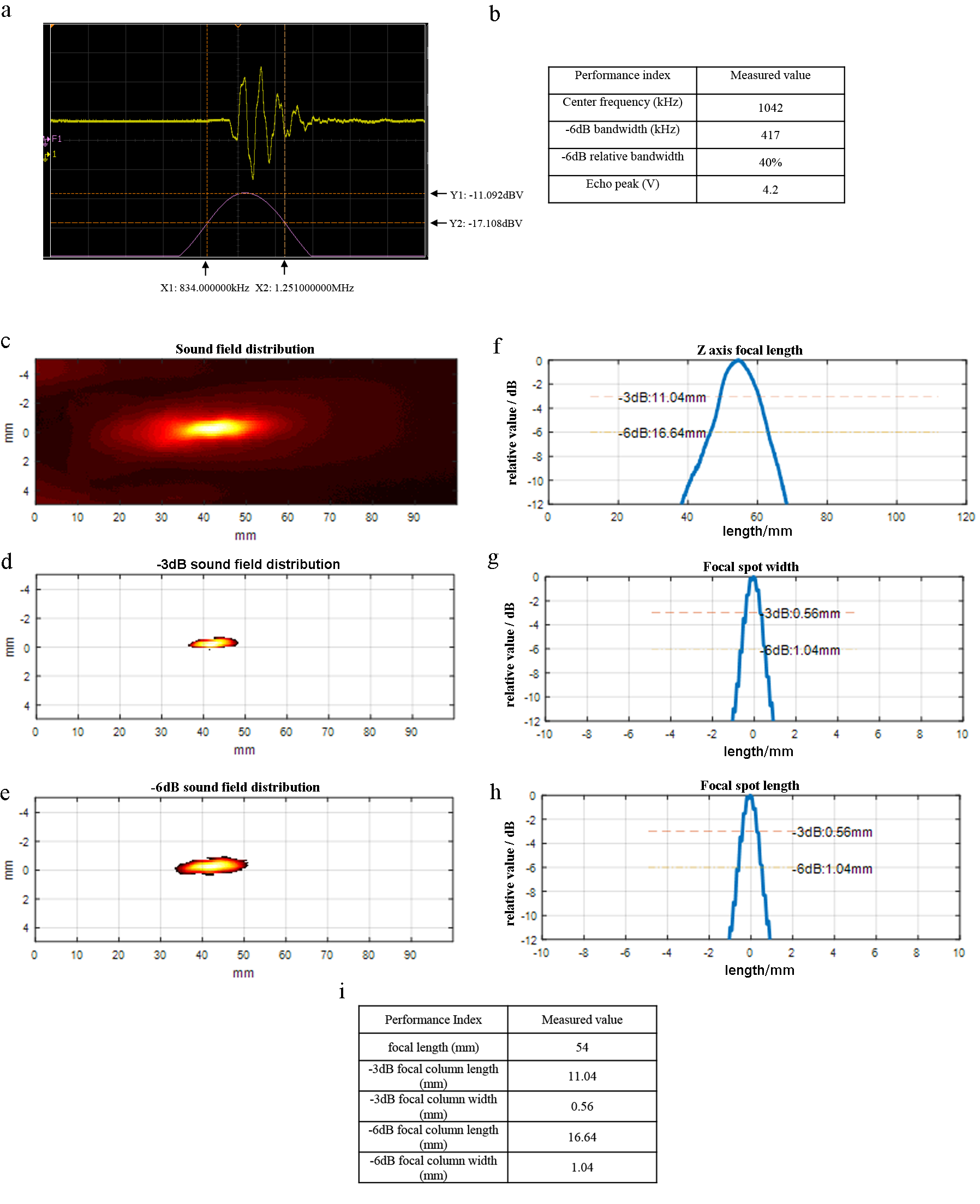

Supplement: Supplementary Materials — Section S1. Figs. S1 to S8. Table S1. [file research.0130.f1.zip › rev-Supplemental Fig. S7.tif]

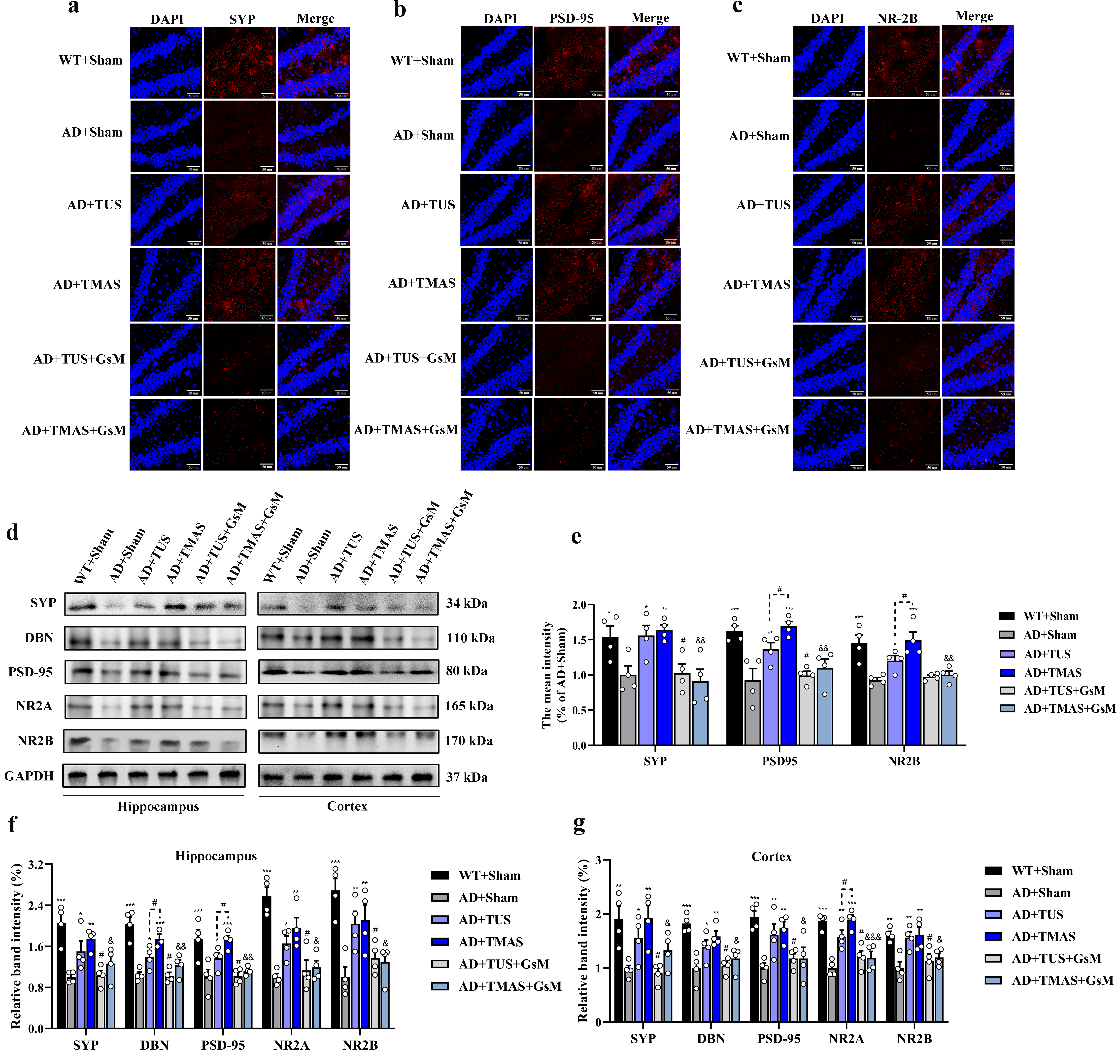

Supplement: Supplementary Materials — Section S1. Figs. S1 to S8. Table S1. [file research.0130.f1.zip › Supplemental Fig. S1.tif]

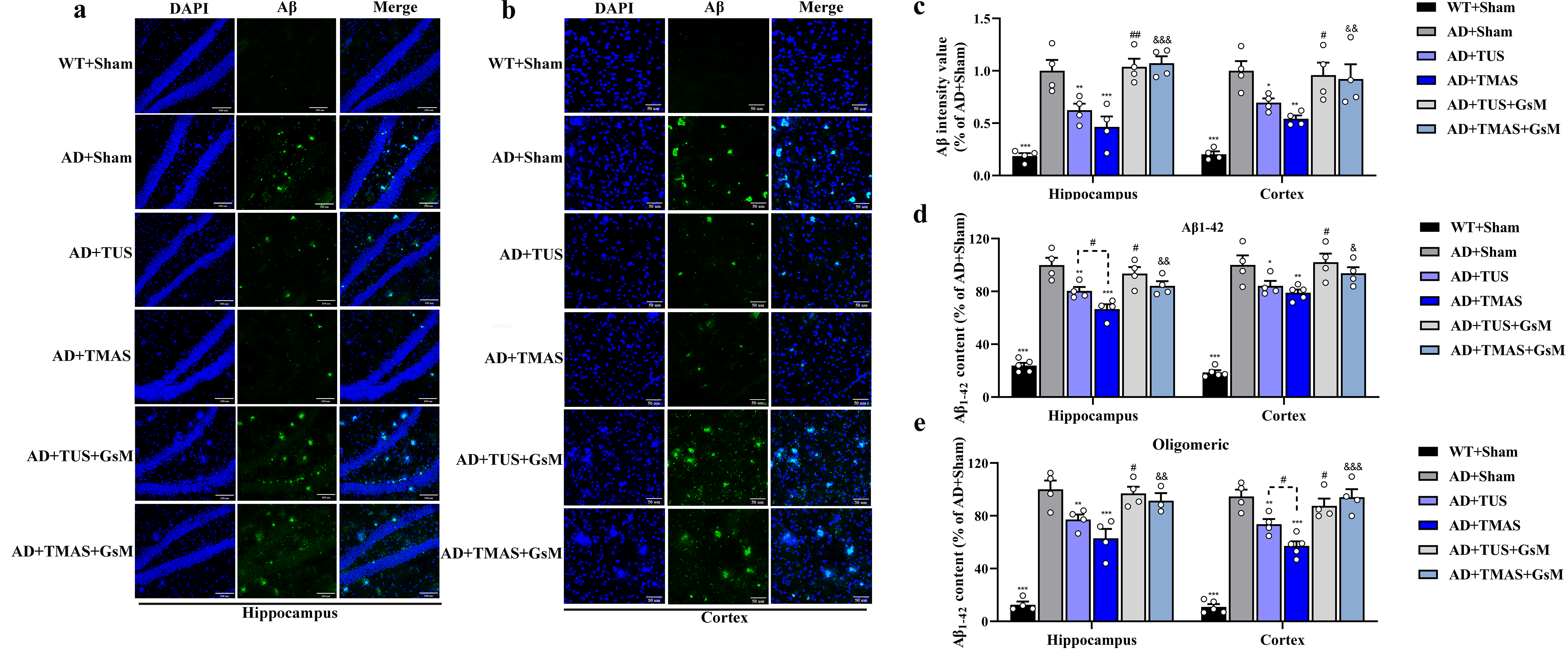

Supplement: Supplementary Materials — Section S1. Figs. S1 to S8. Table S1. [file research.0130.f1.zip › Supplemental Fig. S3.tif]

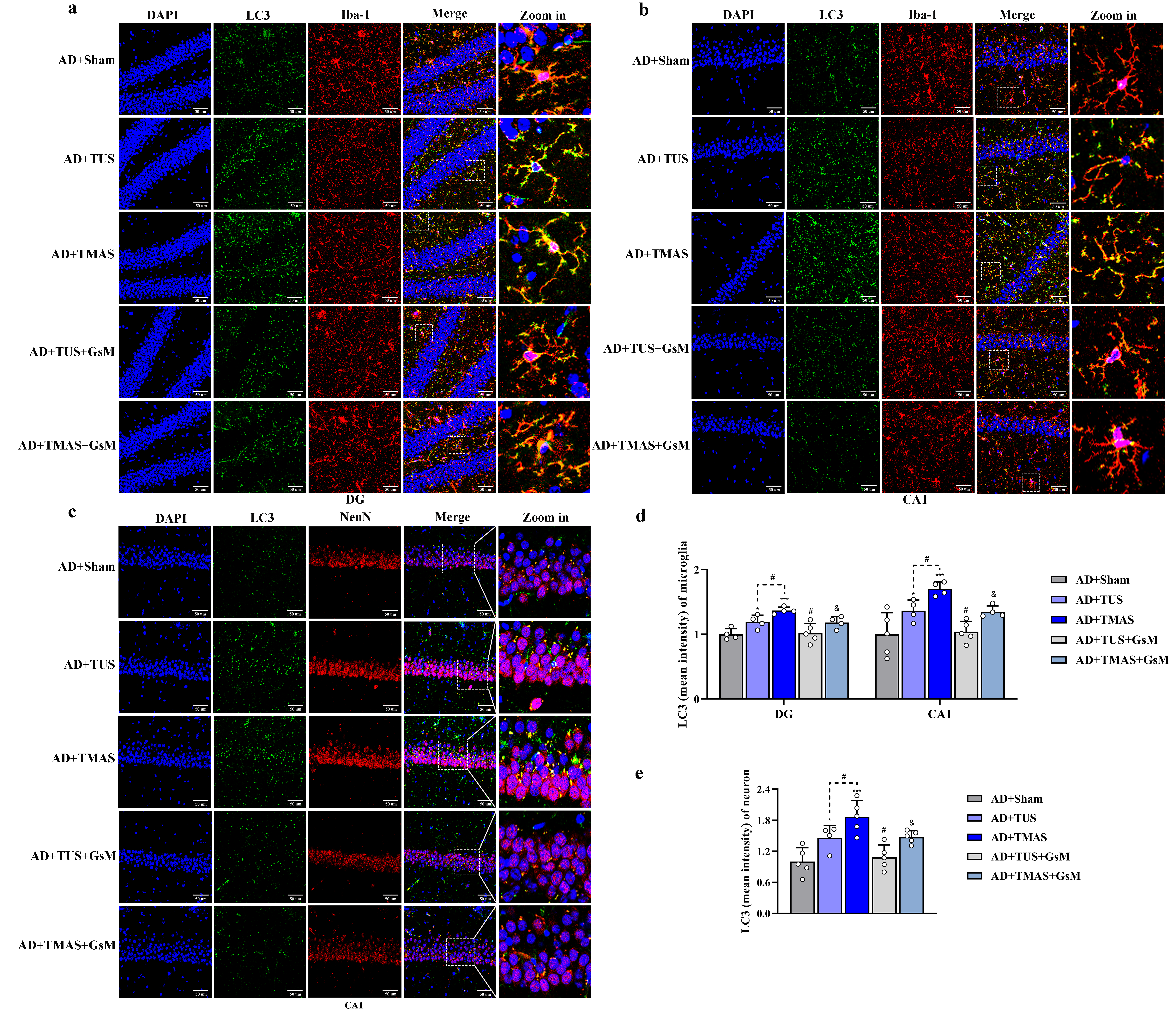

Supplement: Supplementary Materials — Section S1. Figs. S1 to S8. Table S1. [file research.0130.f1.zip › Supplemental Fig. S4.tif]

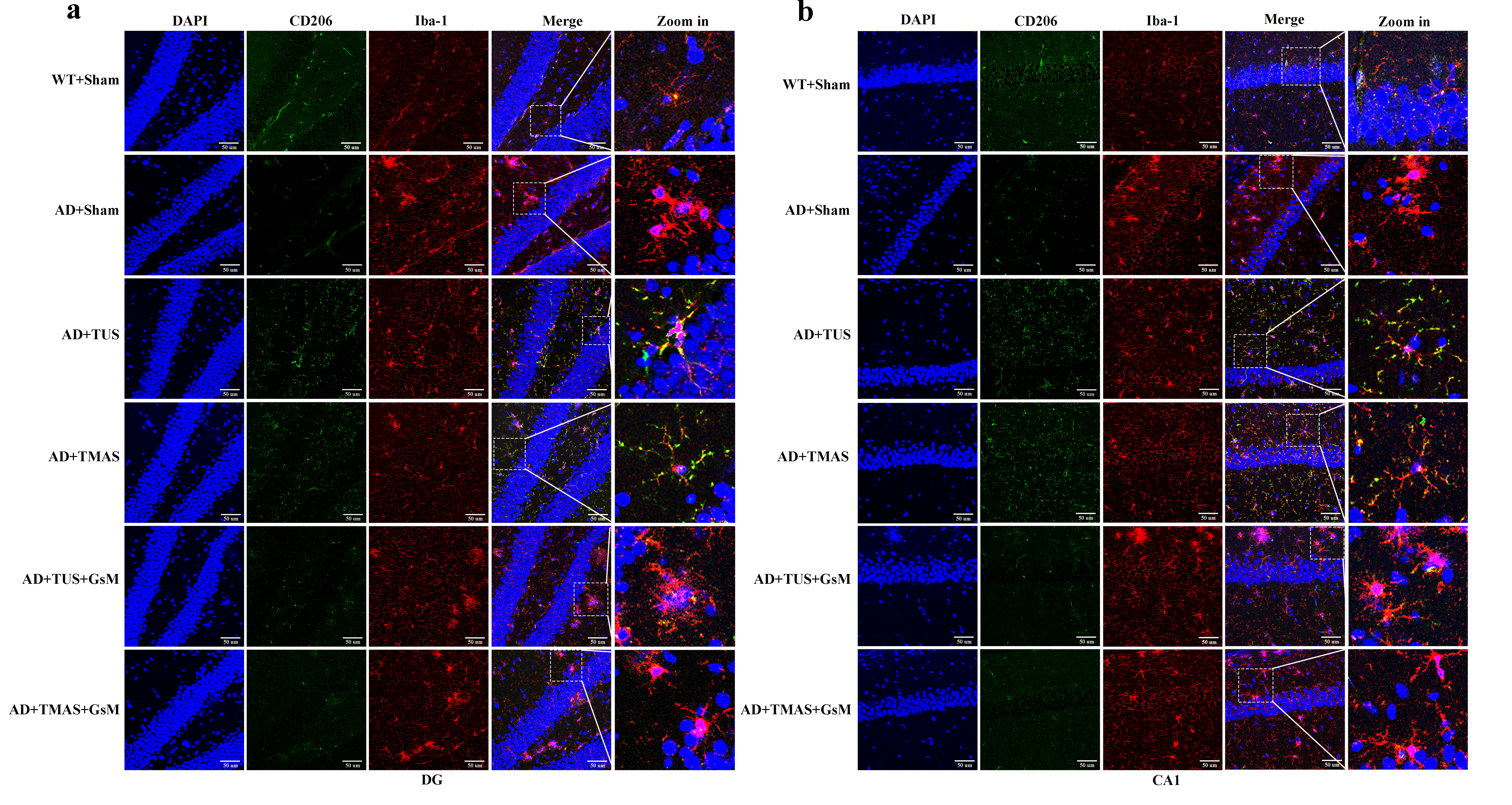

Supplement: Supplementary Materials — Section S1. Figs. S1 to S8. Table S1. [file research.0130.f1.zip › Supplemental Fig. S5.tif]

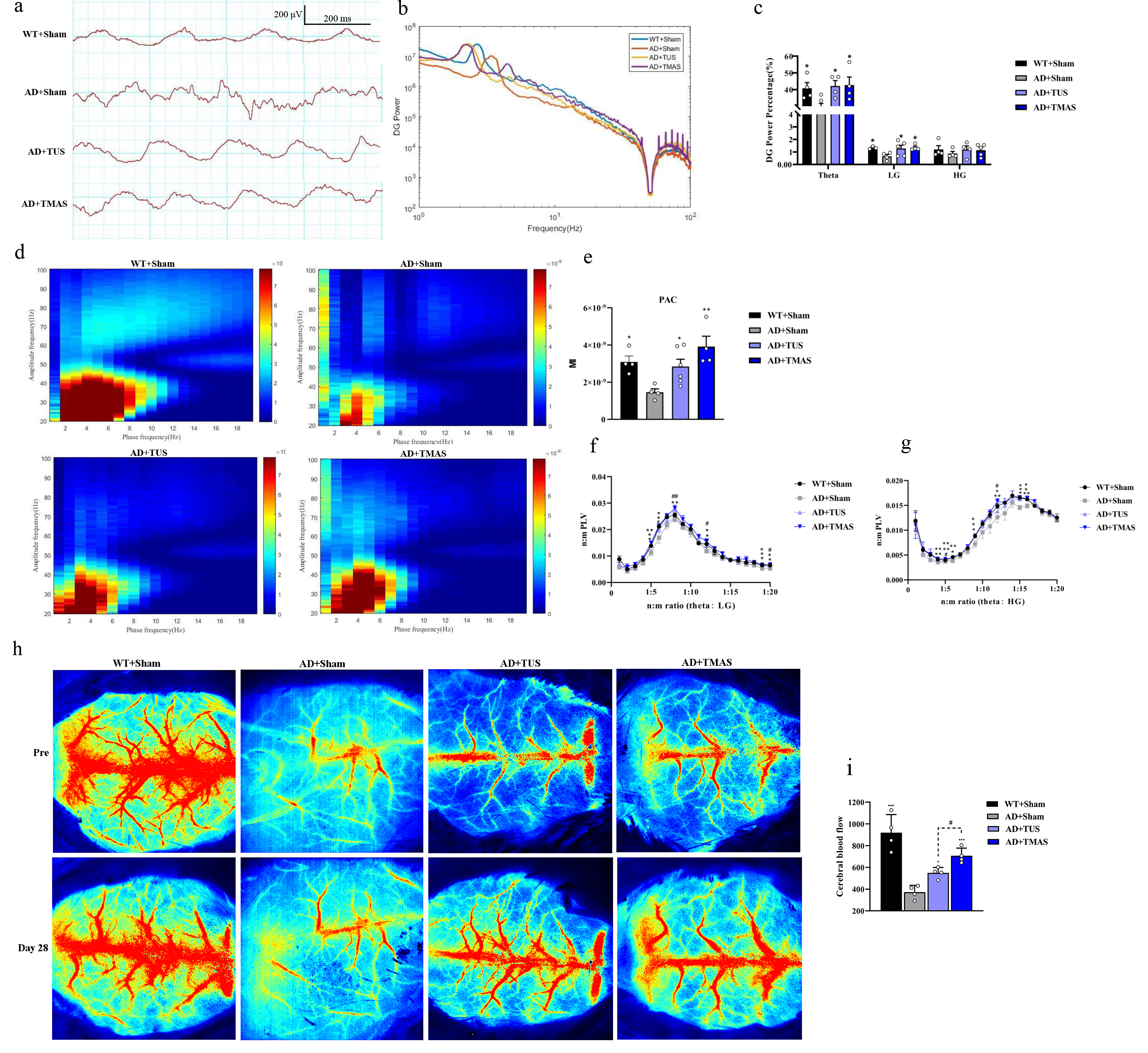

Supplement: Supplementary Materials — Section S1. Figs. S1 to S8. Table S1. [file research.0130.f1.zip › Supplemental Fig. S6..tif]
